# Semaphorin 4C Plexin-B2 signaling in peripheral sensory neurons is pronociceptive in a model of inflammatory pain

**DOI:** 10.1038/s41467-017-00341-w

**Published:** 2017-08-02

**Authors:** Eszter Paldy, Manuela Simonetti, Thomas Worzfeld, Kiran Kumar Bali, Lucas Vicuña, Stefan Offermanns, Rohini Kuner

**Affiliations:** 1 Institute of Pharmacology, Heidelberg University, Im Neuenheimer Feld 366, Heidelberg, 69120 Germany; 2 Institute of Pharmacology, Marburg University, Karl-von-Frisch-Straβe 1, Marburg, 35043 Germany; 3 Department of Pharmacology, Max-Planck-Institute for Heart and Lung Research, Ludwigstrasse 43, Bad Nauheim, 61231 Germany

## Abstract

Semaphorins and their transmembrane receptors, Plexins, are key regulators of axon guidance and development of neuronal connectivity. B-type Plexins respond to Class IV semaphorins and mediate a variety of developmental functions. Here we report that the expression of Plexin-B2 and its high-affinity ligand, Sema4C, persists in peripheral sensory neurons in adult life and is markedly increased in states of persistent pain in mice. Genetic deletion of Sema4C as well as adult-onset loss of Plexin-B2 leads to impairment of the development and duration of inflammatory hypersensitivity. Remarkably, unlike the neurodevelopmental functions of Plexin-B2 that solely rely on Ras signaling, we obtained genetic and pharmacological evidence for a requirement of RhoA-ROCK-dependent mechanisms as well as TRPA1 sensitization in pronociceptive functions of Sema4C-Plexin-B2 signaling in adult life. These results suggest important roles for Plexin-B2 signaling in sensory function that may be of therapeutic relevance in pathological pain.

## Abstract (web summary)

Semaphorins and their receptors are involved in neurodevelopment, but their functions in the adult nervous system are not fully understood. This study finds that semaphorin 4C and its receptor Plexin B are expressed in sensory neurons and are pronociceptive in a mouse model of inflammatory pain.

## Introduction

Amongst plexins, the Plexin-A family and their high-affinity ligands, which include Class V and Class VI semaphorins, as well as Class III semaphorins, which act indirectly with the help of neuropilins, have been largely studied with respect to their functions during development. Recent studies have also revealed insights into the expression and functions of the Plexin-B family receptors and their ligands in the Class IV semaphorin family. Plexin-B1, the prototypic member of the plexin-B family, binds Sema4D^1^, whereas Sema4C is a high-affinity ligand for Plexin-B2^1^; recent studies have also implicated Sema4G, Sema4A and Sema4B as ligands for Plexin-B2^2–4^. Following binding with Sema4 family ligands, which are anchored in the cell membrane or released in soluble form, B-type plexins activate a distinct set of intracellular signaling cascades that play non-redundant and highly context-dependent roles in important cellular functions. We and others have demonstrated functional interactions of Plexin-B proteins with a family of neuronally expressed Rho-guanine exchange factors, resulting in activation of the small GTPase RhoA, and negative modulation of other Rho family GTPases, including Rac and R-Ras is also prevalent in a variety of cell types^5–8^. Moreover, B-type plexins physically interact with the receptor tyrosine kinases (RTK) Met and ErbB-2, resulting in their activation^9, 10^.

Recent studies have uncovered an emerging role for Plexin-B family receptors in key functions in adult life as well as in disease pathogenesis, such as in tumor angiogenesis, growth and metastasis, immune system regulation and inflammation^11–15^. In the nervous system; however, Plexin-B signaling has been functionally investigated primarily in the patterning of the developing brain^1, 16–19^. In contrast to the brain, little is known about B-type plexins in the peripheral nervous system. Importantly, although we and others have extensively studied their developmental roles in neurons^1, 8, 17, 20^, the functions of B-type plexins in mature neurons have not been studied so far. We have previously reported the expression of Plexin-B family members in peripheral sensory neurons of the dorsal root ganglia (DRG)^21^. However, nothing has been reported on their expression or function in the adult peripheral nervous system so far. Because Plexin-B proteins have been recently reported to be expressed in adult organs and implicated in modulating a variety of key functions, we addressed whether the expression of Plexin-B members in the DRG persists in adulthood, and whether it is regulated in states of pathological function of DRG neurons, such as inflammatory pain.

Here we report a role for Plexin-B2-Sema4C signaling in nociceptive sensitization and persistent pain states. We employed mice constitutively lacking Sema4C and generated novel conditional sensory neuron-specific mutants lacking Plexin-B2 either with a perinatal onset or adult-onset. We observed that Sema4C, signaling via Plexin-B2, markedly impacts on the sensitivity of mature peripheral sensory neurons, resulting in nociceptive hypersensitivity; remarkably, our data reveal that Plexin-B2-mediated RhoA signaling in adult neurons, which has not been reported and associated with a key in vivo function so far, specifically mediates inflammatory hypersensitivity. These results uncover a function for Semaphorin-Plexin-B signaling in persistent inflammatory pain.

## Results

### Plexin-B2 is expressed in adult peripheral sensory neurons

**Fig. 1:**
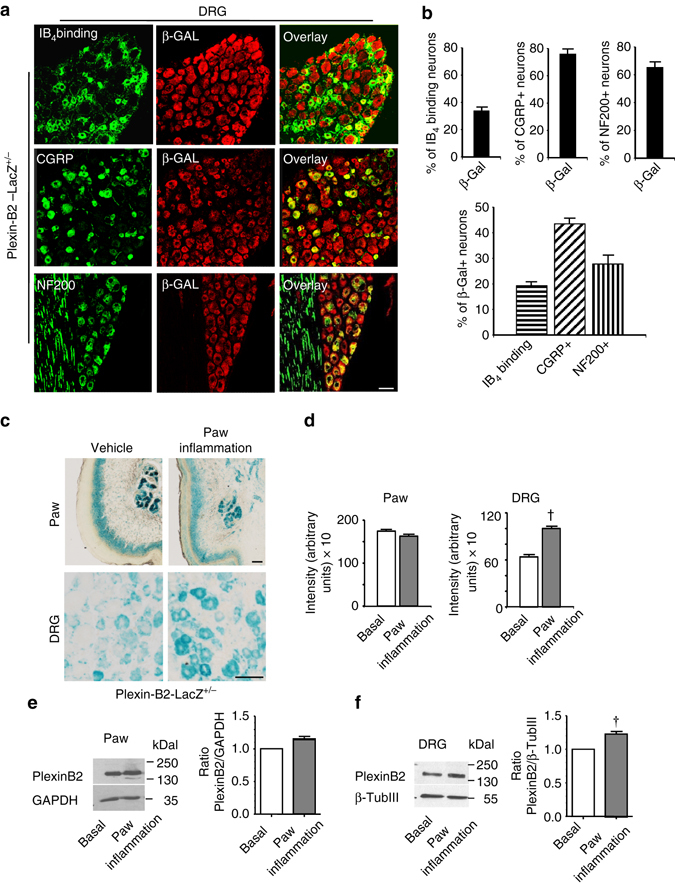
Analysis of Plexin-B2 expression in sensory neurons of the dorsal root ganglia (DRG) in adult mice and its regulation in inflammatory pain. **a**, **b** Expression of *plxnb2* via immunofluorescence analysis of β-galactosidase in adult DRG in respective LacZ reporter knock-in mice. Typical examples **a** and quantitative analysis **b** of the distribution of DRG cell types expressing *plxnb2* via co-immunolabeling with marker proteins (*n* = 10–20 sections/group taken from at least three different mice/group). *Scale bar*, 50 µm. **c**, **d** Typical examples **c** and quantitative summary **d** of LacZ staining demonstrating *plxnb2* expression in adult Plexin-B2-LacZ^+/−^ mice following intraplantar injection of either vehicle (control) or Complete Freund´s Adjuvant (CFA) stimulating inflammatory pain (*n* = 20–30 sections/group taken from at least 3 different mice/group). *Scale bars* represent 50 µm. In **d**, Student’s *t*-test (two sides) was performed. *P* < 0.05 indicated by ^†^ as compared to basal. In all diagrams data are represented as mean, whereas error bars represent s.e.m. **e**, **f** Examples (*left*) and densitometric quantifications (*right*) of western blot analysis of PlexinB2 expression in paw tissue **e** or L3-L4 DRGs **f** in naive mice or at 24 h after intraplantar injection of CFA. Data are represented as fold-increase of the ratio of Plexin-B2 over housekeeping gene signal. All data are presented as mean ± s.e.m. *n* = 8 for paw tissues, *n* = 9 for DRGs. *P* < 0.05 indicated by ^†^ as compared to basal

We utilized transgenic mice expressing β-galactosidase under the endogenous promoter of the *plxnb2* gene (Plexin-B2-LacZ) to map the expression of Plexin-B2 in sensory neurons of the DRG. In adult life, Plexin-B2 was observed to be expressed very broadly across the DRG and double immunohistochemistry for β-galactosidase (β-GAL) and markers of different cell populations in the DRG revealed that more than 70% of peptidergic putative nociceptors and ~65% of large-diameter myelinated neurons expressed β-GAL, but only ~30% non-peptidergic putative nociceptors expressed β-GAL in Plexin-B2-LacZ mice (examples in Fig. 1a and quantitative summary in Fig. 1b). β-GAL-expressing cells comprised of largely peptidergic putative nociceptors (nearly 50%), 20% non-peptidergic putative nociceptors and about 30% large-diameter myelinated neurons (Fig. 1b, *lower panel*). Negative controls for β-Gal immunostaining are shown in Supplementary Fig. 1B. In LacZ histological staining experiments in Plexin-B2-LacZ mice, we observed that LacZ staining in the DRG and as well as in the paw skin, particularly in the keratinocyte layer (Fig. 1c, *left panel*). In Plexin-B1-LacZ mice, in contrast, LacZ staining was neither localized in adult DRG nor in the paw skin (Supplementary Fig. 1A). In an independent series of experiments, we tested an antibody against Plexin-B2 in immunohistochemistry on DRG neurons (Supplementary Fig. 1C), which was validated for specificity in tissue derived from Plexin-B2 knockout mice (Supplementary Fig. 1D; full uncropped immunoblots are shown in Supplementary Fig. 8J) and observed that 100% of DRG neurons immunoreactive for endogenous Plexin-B2 also expressed LacZ in PB2-LacZ reporter mice (Supplementary Fig. 1C). Moreover, 97 ± 2.8 % of LacZ-expressing neurons were immunoreactive for Plexin-B2 in Plexin-B2-LacZ reporter mice (Supplementary Fig. 1C). This very high level of co-localization indicates that the LacZ reporter indeed faithfully recapitulates the expression of Plexin-B2.

LacZ histochemical staining is a sensitive indicator for promoter activity in reporter mice and also enables quantitative comparisons in expression levels. Although LacZ staining intensity can vary across experiments, analysis of intensity of the LacZ staining product within an experiment enables comparison of control and test samples^22^. Using this technique, we addressed whether the expression of Plexin-B2 in sensory neurons is induced in pathological states associated with increased nociceptive drive, such as in inflammatory pain. Upon inducing unilateral hindpaw inflammation via intraplantar injection of Complete Freund’s adjuvant (CFA) in Plexin-B2-LacZ reporter mice, we found that LacZ staining intensity significantly increased in neurons of the ipsilateral L3-L4 DRGs of inflamed mice, but remained unchanged in the inflamed paw skin (examples in Fig. 1c and quantitative summary of LacZ intensity over unit area of the DRG in Fig. 1d). In the above experiments, all sections from all mice belonging to both groups were stained within the same staining and development round and microscopic analyses were performed within the same session keeping all parameters equal across all images. Moreover, we independently confirmed that Plexin-B2 is upregulated in the DRGs of inflamed mice, but not inflamed paw, as compared to control mice via Western blot analysis (Fig. 1e, f; full uncropped immunoblots are shown in Supplementary Fig. 8A, B).

### Plexin-B2 deletion affects basal nociception and acute pain

**Fig. 2:**
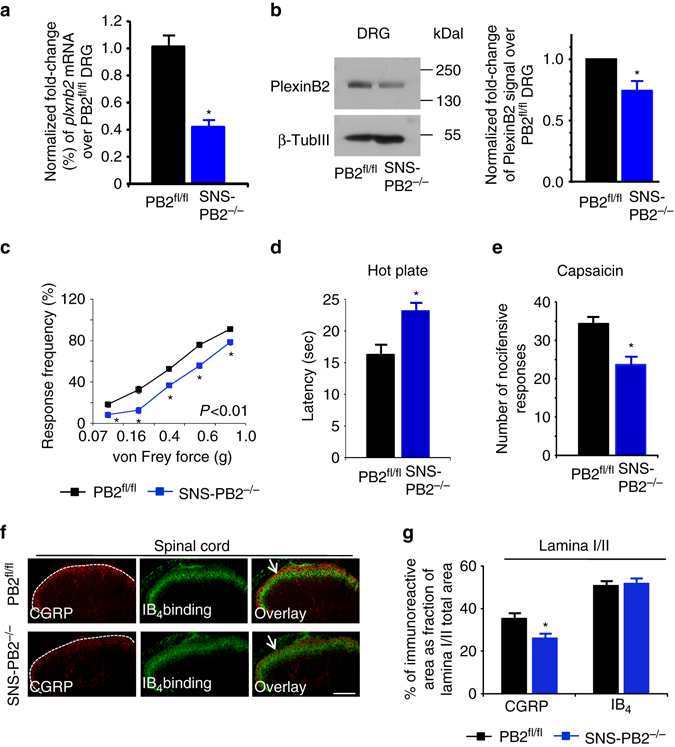
Morphological and functional analyses in mice with early onset loss of Plexin-B2 in peripheral sensory neurons. **a** Quantitative RT-PCR for *plxnb2* expression in the DRG of adult SNS-PB2^−/−^ or PB2^fl/fl^ mice (*n* = 4 for PB2^fl/fl^ mice and *n* = 6 for SNS-PB2^−/−^ mice). **b** Examples (*left*) and densitometric quantifications (*right*) of western blot analysis of PlexinB2 in lysates of L3-L4 DRGs from SNS-PB2^−/−^ mice or their control littermates PB2^fl/fl^. Data represent fold changes of the ratio of Plexin-B2 over β-TubulinIII signal and shown as mean ± s.e.m. *n* = 5 mice/group, *P* < 0.05. **c**–**e** Behavioral analysis of sensitivity to somatosensory nociceptive and non-nociceptive stimuli tested in the hindpaw SNS-PB2^−/−^ mice or their control PB2^fl/fl^ littermates. **c** Basal mechanical sensitivity measured as frequency of withdrawal responses to five applications each of von Frey filaments of varying force (*n* = 12 mice/group). **d** Analysis of latency of paw withdrawal to 50 °C heat (*n* = 10 mice/group). **e** Analysis of nocifensive behavior evoked by intraplantar injection of the algogen, capsaicin (*n* = 10 mice/group). **f**, **g** Typical examples **f** and quantitative summary **g** of immunohistochemical determination of the spinal targeting of CGRP-positive and IB_4_-binding nociceptors (*n* = 14–18 sections/group taken from three different mice/group). *Scale bar*, 100 µm. ANOVA repeated measures was performed in panel c comparing entire curves with each other, followed by Tukey’s test. In **a**, **b**, **d**, **e** and **g**, Student’s *t*-test was performed. In all panels, *P* < 0.05 indicated by * as compared to the corresponding control groups. Error bars represent s.e.m

To address the function of Plexin-B2 in modulation of pain sensitivity, we generated mice lacking Plexin-B2 conditionally primarily in nociceptive neurons of the DRG (SNS-PB2^−/−^ mice; Supplementary Fig. 2A) by employing SNS-Cre mice^23^, which we and others have characterized in details in previous studies to yield recombination starting prenatally^20, 22^. Loss of Plexin-B2 expression in the DRG was confirmed by quantitative real-time PCR (qRT-PCR) analysis (Fig. 2a) as well as via western blotting with the anti-Plexin-B2 antibody (Fig. 2b; full uncropped immunoblots are shown in Supplementary Fig. 8C). Adult SNS-PB2^−/−^ mice showed normal body weight and no overt phenotypic abnormalities (Supplementary Fig. 2B). Analyses of adult mice in sensory behavioral tests revealed significant basal hyposensitivity to somatosensory nociceptive stimuli in SNS-PB2^−/−^ mice. This was evident as reduced sensitivity to mechanical force via punctate von Frey filaments applied to the plantar surface^24^ (Fig. 2c) as well as increased response latency in the hot plate test^24^ (Fig. 2d) in SNS-PB2^−/−^ mice as compared to PB2^fl/fl^ mice. Moreover, SNS-PB2^−/−^ mice demonstrated significantly attenuated nocifensive responses, such as biting and licking of the paw upon intraplantar injection of the algogen, capsaicin (Fig. 2e). There were no obvious defects found with respect to motor coordination (Supplementary Fig. 2C). We also did not find any sex differences with respect to nociception and hypersensitivity in any of the groups in this study.

However, these defects in basal nociceptive sensitivity were associated with morphological abnormalities in peripheral nociceptive neurons, underscoring a developmental role for Plexin-B2. In particular, SNS-PB2^−/−^ mice showed a significant reduction in the density of projections of peptidergic putative nociceptive afferents into the spinal cord (Fig. 2f, g) as well as peripherally in the skin (Supplementary Fig. 2D, E). Spinal projections of non-peptidergic putative nociceptive neurons were normal in SNS-PB2^−/−^ mice (Fig. 2g). Negative controls for immunostaining are shown in Supplementary Fig. 1E. Moreover, SNS-PB2^−/−^ mice demonstrated a significantly reduced number of peptidergic putative nociceptive neurons in the DRG as compared to wild-type mice, whereas the number of non-peptidergic putative nociceptive neurons and large diameter NF-200-expressing neurons was unchanged (Supplementary Fig. 2F). Owing to these developmental defects, we surmised that SNS-PB2^−/−^ mice may be of limited value in addressing the role of Plexin-B2 in adult sensory neurons.

### Plexin-B2 deletion in adult DRG affects inflammatory pain

**Fig. 3:**
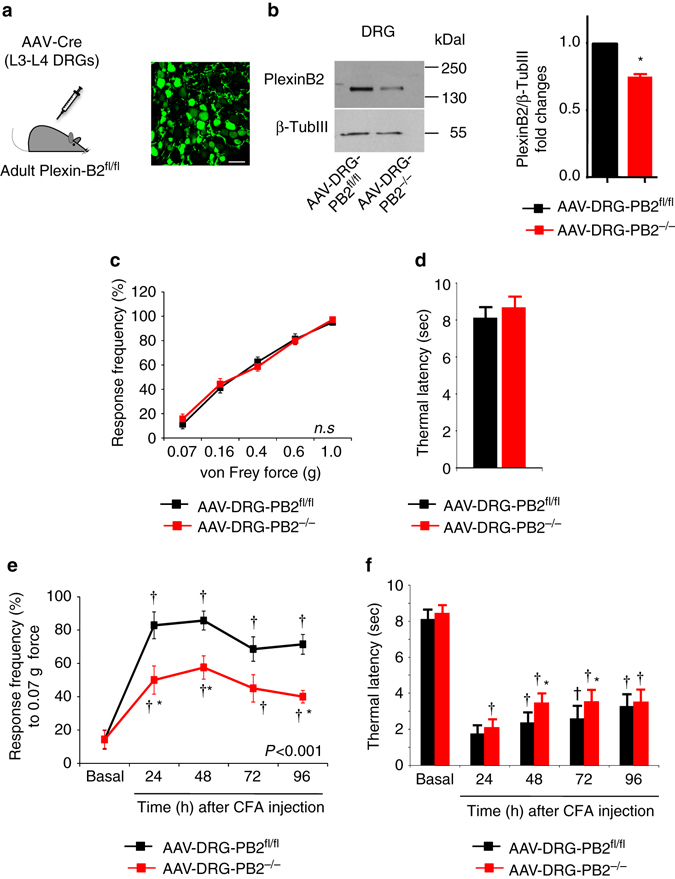
Defects in inflammatory hypersensitivity in mice lacking with adult-onset conditional deletion of Plexin-B2 in the DRG. **a** Schematic representation (*left*) and a typical example of adeno-associated viral (AAV) delivery of Cre recombinase or GFP (*control, right*) to sensory neurons via direct injection in L3-L4 DRGs of adult PB2^fl/fl^ mice. *Scale bar*, 75 µm. **b** Examples (*left*) and densitometric quantifications (*right*) of western blot analysis of Plexin-B2 in lysates of L3-L4 DRGs from AAV-DRG-PB2^−/−^ mice or their control AAV-DRG-PB2^fl/fl^ littermates. Data are represented as fold changes of the ratio of Plexin-B2 over β-TubulinIII signal; *n* = 4 mice/group, *P* < 0.05. **c**, **d** Analysis of basal mechanical sensitivity to von Frey filaments **c** and basal thermal sensitivity to plantar application of radiant heat (Plantar test, **d**) in mice lacking Plexin-B2 conditionally in adult DRG (AAV-DRG-PB2^−/−^) and their corresponding AAV-DRG-PB2^fl/fl^ controls expressing GFP instead of Cre recombinase (*n* = 7 mice/group). **e**, **f** Analysis of inflammatory mechanical hypersensitivity **e** and thermal hyperalgesia **f** following hindpaw CFA injection in AAV-DRG-PB2^−/−^ mice and in their controls. In **e**, frequency of paw withdrawal in response to application of 0.07 g force via a von Frey filament is shown (*n* = 7 mice/group). ANOVA for repeated measures in **c**, **e** and **f** followed by Tukey’s test and Student’s *t*-test in **b** and **d** were performed. In panel f, *P* < 0.001 upon comparing the entire curves with each other. In all panels, *P* < 0.05 indicated by * as compared to the corresponding control groups and by ^†^ as compared to basal. Error bars represent s.e.m

To circumvent potential confounding results arising from developmental abnormalities, we induced an adult-onset genetic deletion of Plexin-B2 by transducing recombinant adeno-associated virions (rAAV1/2) expressing the Cre recombinase as well as GFP into the L3 and L4 DRGs of PB2^fl/fl^ mice (AAV-DRG-PB2^−/−^ mice; see Fig. 3a for strategy and an example of AAV-mediated GFP expression in adult DRG). Controls comprised of PB2^fl/fl^ mice injected with rAAV-GFP (i.e., no Cre; AAV-DRG-PB2^fl/fl^). This is a highly established procedure, which we have previously characterized and published^25–27^. We have previously reported that with the use of viral serotypes employed here, one achieves a neuron-specific, stable and broad expression, without affecting gene expression in satellite cells, Schwann cells or blood vessels in the DRG^26^. We observe that ~82 ± 6% neurons are transduced, broadly spanning small-diameter and large diameter populations and identified markers of nociceptive and non-nociceptive neurons. Moreover, the lack of laminectomy renders this a minimally invasive procedure and we have observed a lack of toxicity at the time of behavioral testing at 4 weeks post viral injection^26^. In all experiments, post-mortem analysis of GFP expression in L3-L4 DRGs was verified and only mice showing viral expression in at least 75–80% of DRG neurons were included in the analysis.

Western blot analysis on L3-L4 DRGs demonstrated significant decrease in expression of PlexinB2 in AAV-DRG-PB2^−/−^ mice as compared to AAV-DRG-PB2^fl/fl^ mice (examples shown in Fig. 3b *left* and quantitative summary shown in Fig. 3b *right* panel; full uncropped immunoblots are shown in Supplementary Fig. 8D). AAV-DRG-PB2^−/−^ mice did not reveal any abnormalities in basal sensitivity to mechanical tactile function, mechanical nociceptive responses and responses to heat as compared to AAV-DRG-PB2^fl/fl^ mice (Fig. 3c, d), indicating a lack of requirement for Plexin-B2 signaling in basal nociception.

However, acute as well as long-lasting mechanical hypersensitivity following paw injection of CFA was significantly attenuated in AAV-DRG-PB2^−/−^ mice as compared to controls (response frequency to individual filament strength is shown in Fig. 3e and cumulative responses to all von Frey filaments tested are shown in Supplementary Fig. 3A. In contrast to this marked phenotype with the mechanical modality, inflammatory heat hyperalgesia developed normally for the most part in AAV-DRG-PB2^−/−^ mice (Fig. 3f); however, significant differences to control mice were evident at 48 and 72 h post CFA. The magnitude of paw inflammation was comparable across both groups, as confirmed via quantification of neutrophil infiltration via Gr-1 expression in the inflamed paw (vehicle control, examples and quantification shown in Supplementary Fig. 3B, C). Thus, adult-onset loss of Plexin-B2 in peripheral sensory neurons was functionally associated with reduced mechanical inflammatory hypersensitivity, but not to deficits in basal nociception.

### Sema4C plays a role in inflammatory hypersensitivity

**Fig. 4:**
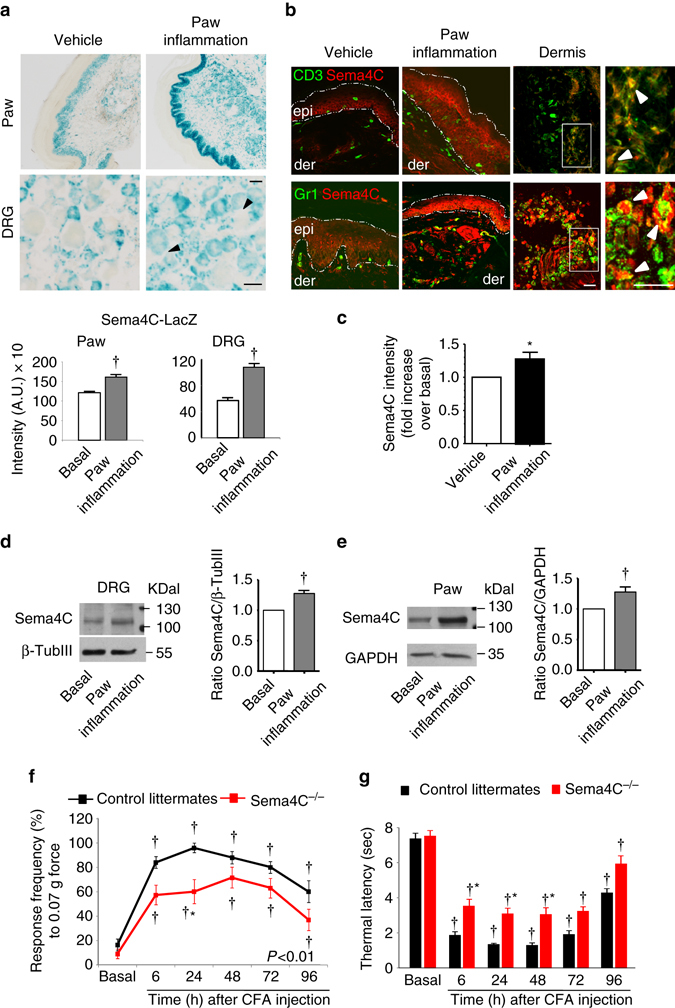
Sema4C is expressed in adult DRG and paw skin and plays a functional role in inflammatory pain. **a** Expression of *Sema4c* via β-galactosidase (LacZ) staining in adult DRG and plantar paw skin using LacZ reporter knock-in mice. Typical examples (*upper*) and quantitative summary (panel) of LacZ staining in the basal state or following CFA-induced paw inflammation. *Scale bars*, 50 µm. **b** Expression of Sema4C and its co-localization with immune cells markers via double immunofluorescence staining in plantar paw skin of mice at 24 h after vehicle or CFA injection, using anti-Sema4C antibody and antibodies against immune cells (CD3 to mark infiltrating T cells, *upper*; or GR-1 to target macrophages, *lower*). Higher magnification views of dermis are shown on extreme right to illustrate cells showing co-localization (*arrowheads*). *Scale bars*, 25 µm. **c** Quantitative measurement of intensity of Sema4C immunoreactivity paw tissue 24 h after intraplantar injection of vehicle or CFA; *n* = 3 mice/group. **d**, **e** Examples (*left*) and densitometric quantifications (*right*) of western blot analysis of Sema4C signal in lysates of L3-L4 DRGs **d** or paw tissue **e** 24 h after intraplantar CFA injection; *n* = 8 for DRGs, *n* = 9 for paw tissues. **f** Analysis of inflammatory mechanical hypersensitivity following hindpaw CFA injection in mice lacking Sema4C (Sema4C^−/−^) and their wild-type littermates. Frequency of paw withdrawal in response to application of 0.07 g force via a von Frey filament is shown. **g** Changes in paw response latency to radiant heat following CFA injection in Sema4C^−/−^ mice and their wild-type controls littermates. *n* = 5 (Sema4C^−/−^ mice) and *n* = 7 (wild-type littermates) mice/group. Student’s *t*-test was performed in **a**–**e** and two-way ANOVA for repeated measures followed by Tukey’s test was performed in **f** and **g**. In **f**, *P* < 0.01 upon comparing the entire curves with each other. In all panels, *P* < 0.05 indicated by * as compared to the corresponding control groups and by ^†^ as compared to basal. Error bars represent s.e.m

We then analyzed expression and function of Sema4C in the context of hypersensitivity modulation. Using mice expressing *LacZ* under the control of the *sema4c* promoter (Sema4C-LacZ mice), we observed that Sema4C is expressed in adult DRG neurons as well as in the paw skin, including the keratinocyte layer in which we had observed Plexin-B2 expression (Fig. 4a, *left*). Moreover, similar to Plexin-B2, LacZ staining intensity was markedly increased in the DRG of Sema4C-LacZ mice with CFA-induced paw inflammation—particularly, large-diameter neurons (arrowheads in Fig. 4a) and satellite cells demonstrated LacZ expression in inflamed Sema4C-LacZ mice (Fig. 4a, *right*; quantification over unit area of the DRG in Fig. 4a, *lower*; all LacZ staining experiments and intensity measurements performed within the same round of analyses including mice of both groups). Interestingly, however, unlike Plexin-B2, LacZ staining intensity was also significantly upregulated in the paw skin in the keratinocyte layer as well as the dermis and subcutaneous tissue, which are occupied by infiltrating immune cells (examples in Fig. 4a *upper panel* and quantitative summary in Fig. 4a *lower panel*). We then confirmed expression of Sema4C in the skin with an anti-Sema4C antibody; validation of lack of staining in mice constitutively lacking *sema4c* (Sema4C^−/−^) is shown in Supplementary Fig. 4A. In mice with paw inflammation, increased expression of anti-Sema4C immunoreactivity was observed primarily in the keratinocyte layer, and in some cells that populate the dermis of the inflamed paw (example in Fig. 4b and quantification of signal intensity in Fig. 4c). Co-immunolabelling with anti-CD3 antibody, which labels infiltrating T cells, revealed that T cells mostly populate the deeper parts of the dermis and ~28 ± 4.2 % show co-localization with Sema4C. Moreover, co-staining with an anti-Gr1 antibody, which labels macrophages, revealed co-labeling in about 33 % ± 3.9 of macrophages in more superficial layers just below the keratinocyte layer and 55 ± 5.6 % macrophages located deeper in the dermis (Fig. 4b and Supplementary Fig. 4B, C). Thus, regions of the inflamed plantar skin that harbor terminals of Plexin-B2-expressing sensory neurons as well as some sub-populations of immune cells showed a marked increase in the availability of Sema4C.

Finally, we also confirmed upregulation of Sema4C in the paw skin as well as the L3-L4 DRG of inflamed mice as compared to control mice via Western blotting with the anti-Sema4C antibody (typical example and quantification shown in Fig. 4d, e; full uncropped immunoblots are shown in Supplementary Fig. 8E, F). Supplementary Fig. 4D shows that the bands corresponding to Sema4C protein are missing in lysates derived from Sema4C^−/−^ mice (full uncropped immunoblots are shown in Supplementary Fig. 8K).

Sema4C^−/−^ mice demonstrated normal basal sensitivity to mechanical and thermal stimuli applied to the plantar skin (Fig. 4f, g). This was consistent with a lack of developmental defects in the nociceptive patterning of afferents in Sema4C^−/−^ mice (examples and quantification shown in Supplementary Fig. 4E, F). Interestingly, similar to mice lacking Plexin-B2 in sensory neurons, Sema4C^−/−^ mice developed inflammatory mechanical hypersensitivity to a markedly lower extent than control littermates (Fig. 4f; cumulative responses over all time points and all von Frey mechanical forces tested are shown in the Supplementary Fig. 5A). Similarly, inflammatory thermal hyperalgesia was significantly less pronounced in Sema4C^−/−^ mice as compared to wild-type control littermates (Fig. 4g).

### Sema4C-RhoA-ROCK signaling induces nociceptive sensitization

**Fig. 5:**
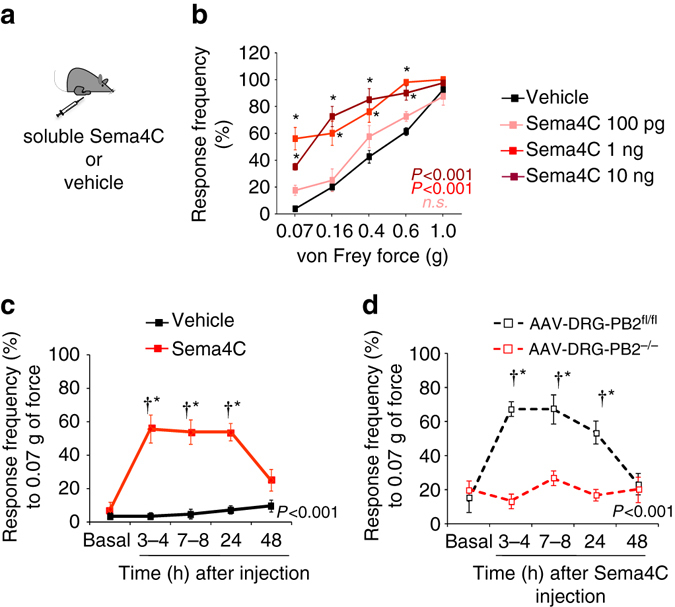
Intraplantar paw injection of Sema4C causes rapid nociceptive sensitization. **a** A schematic representation of unilateral intraplantar Sema4C application in adult wild-type mice. **b** Analysis of mechanical sensitivity following a single injection of Sema4C at various doses or vehicle. Response frequency to five applications of von Frey filaments exerting graded force measured at 3 h following Sema4C injection is shown. **c** Time-course analyses of the effect of a single intraplantar dose of 1 ng Sema4C on sensitivity to 0.07 g of von Frey hair force. **d** Frequency of paw withdrawal in response to application of 0.07 g force via a von Frey filament upon single intraplantar dose of 1 ng Sema4C in mice lacking Plexin-B2 conditionally in adult DRG (AAV-DRG-PB2^−/−^) and their corresponding AAV-DRG-PB2^fl/fl^ controls (*n* = 7 mice/group). In all panels, two-way ANOVA for repeated measures followed by Tukey’s test was performed, and *P* values comparing entire curves are represented. *P* < 0.05 indicated by * as compared to the corresponding control groups and by ^†^ as compared to basal. Error bars represent s.e.m

Having observed modulation of nociception by endogenously expressed Plexin-B2 as well as Sema4C in adult mice, we then went on to address underlying mechanisms. First, we simulated the upregulation of Sema4C protein observed in the paw skin of inflamed mice by injecting soluble recombinant Sema4C unilaterally in the plantar skin of naive wild-type mice (see Fig. 5a for strategy). We have previously reported on the specific Plexin-B2-activating properties of the soluble fraction of Sema4C^1, 17^. Recombinant Sema4C injected intraplantar into the hindpaw of adult mice dose dependently caused a marked and rapid sensitization towards mechanical von Frey stimuli within about 2–3 h, which lasted until at least 24 h after a single application (Fig. 5b, c); equal quantities of albumin injected as a control protein did not cause sensitization (Supplementary Fig. 6A). A typical response-frequency curve to 0.07 g of force after application of 1 ng Sema4C is shown in Fig. 5c and the integrals of responses over all time points and all von Frey mechanical forces applied are shown in Supplementary Fig. 5B.

**Fig. 6:**
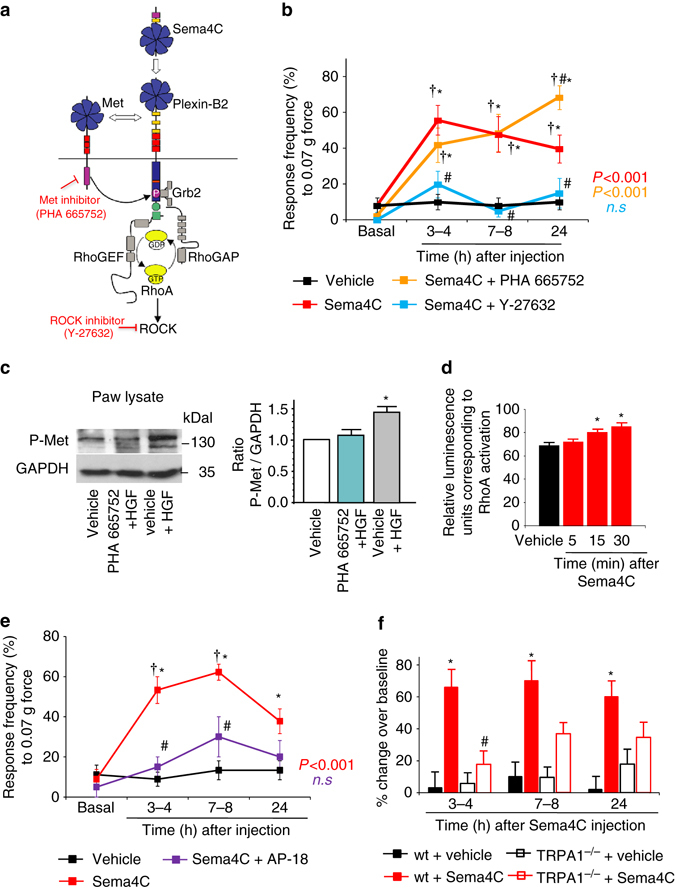
Sema4C activates the RhoA-ROCK pathway in peripheral sensory neurons to induce mechanical hypersensitivity. **a** Schematic representation of the diverse components of Sema4C/Plexin-B2 signaling pathway tested in this study **b** Mechanical hypersensitivity to von Frey stimuli (0.07 g shown) induced by plantar application of Sema4C in the presence or absence of a ROCK 1/2 inhibitor (Y-27632, 2 ng), an inhibitor of Met signaling (PHA 665752, 3.3 μg) or vehicle (*n* = 8 mice/group for Sema4C + Y-27632 treatment, *n* = 9 for Sema4C + PHA 665752 treatment and *n* = 10 each for vehicle or Sema4C treatment). **c** Examples (*left*) and densitometric quantification (*right*) of western blot analysis of Met phosphorylation (P-Met) in paw tissue 15 min after intraplantar injection of recombinant mouse Hepatocyte growth factor (50 ng) in the presence of a Met inhibitor (PHA 665752, 3.3 μg) or vehicle. Data are represented as fold-increase of the ratio of P-Met over GAPDH expression over vehicle-treated group; *n* = 3 independent blots and mice/group. *P* < 0.05 indicated by * as compared to vehicle-treated group. **d** Analysis of active RhoA in cultured DRG neurons upon treatment with Sema4C (150 nM) or vehicle (*n* = 6 independent culture experiments). **e** Analysis of mechanical hypersensitivity induced by plantar application of Sema4C in the presence or absence of a TRPA1 inhibitor (AP-18, 10 nmol) or vehicle (*n* = 4 mice/group for Sema4C + AP-18 treatment, *n* = 9 each for vehicle or Sema4C treatment). Frequency of paw withdrawal in response to application of 0.07 g force via a von Frey filament is shown. **f** Analysis of Sema4C-induced mechanical hypersensitivity in mice lacking TRPA1 (*TRPA1* ^−/−^) and wild-type (wt) controls, expressed as % change over baseline sensitivity (*n* = 8 mice/group). ANOVA for random (**c**) and repeated (**b**, **d**, **e** and **f**) measures followed by Tukey’s test, and *P* values comparing entire curves with each other are represented. In all panels, *P* < 0.05 indicated by * as compared to the corresponding control groups and by ^†^ as compared to basal and by ^#^ as compared to Sema4C-treated group. Error bars represent s.e.m

Importantly, Sema4C failed to induce mechanical hypersensitivity in AAV-DRG-PB2^−/−^ mice, in contrast to control mice (Fig. 5d), demonstrating that this was indeed mediated functionally by Plexin-B2 expressed in peripheral sensory neurons. We then addressed potential contributions of pathways downstream of Plexin-B2 signaling (schematically represented in Fig. 6a). Pharmacological inhibition of the RhoA-ROCK pathway via intraplantar injection of a ROCK inhibitor, Y-27632 (2 ng administered 15 min prior to Sema4C injection) entirely blocked mechanical hypersensitivity elicited by 1 ng Sema4C (Fig. 6b; Supplementary Fig. 6B), whereas inhibition of Met with PHA665752 (3.3 μg) did not influence Sema4C-induced mechanical hypersensitivity (Fig. 6b; Supplementary Fig. 6B). The doses of inhibitors were chosen based on reported effective doses for in vivo usage in other studies^28, 29^ and neither inhibitor affected mechanical sensitivity when administered in the absence of Sema4C (Supplementary Fig. 6B, C). To ascertain that the Met inhibitor was functionally working in the paw tissue upon intraplantar delivery, we analyzed Met phosphorylation after intraplantar injection of the Met agonist, Hepatocyte growth factor (HGF, 50 ng), in presence of PHA665752 (3.3 µg intraplantar) or vehicle. In paw lysates, 15 min after HGF treatment, we observed a marked increase in Met phosphorylation over vehicle-treated paw samples (Fig. 6c; full uncropped immunoblots are shown in Supplementary Fig. 8G), which was abrogated upon pretreatment with PHA665752, indicating that this dose of the Met inhibitor was efficacious under the conditions in which it failed to block Sema4C-induced hypersensitivity (Fig. 6c).

Inhibition of ROCK also abrogated Sema4C-induced thermal hyperalgesia (Supplementary Fig. 6D). In keeping with these in vivo observations, we noted that Sema4C significantly increased the levels of activated endogenous RhoA in comparison with vehicle treatment in cultured DRG neurons (Fig. 6d).

### A role for TRPA1 in Sema4C-induced hypersensitivity

Because these experiments indicated a role for Plexin-B2 signaling in modulating mechanical sensitization, but not basal mechanical sensitivity, we addressed potential contributions of TRPA1, which is one of the ion channels that have been implicated in mechanical sensitization, but not in basal tactile sensation or mechanical pain, in pharmacological as well as genetic studies^30–32^. We observed that intraplantar pretreatment with AP-18 (10 mmol^32^) an inhibitor of TRPA1, significantly reduced mechanical hypersensitivity in response to plantar application of 1 ng Sema4C (Fig. 6e and Supplementary Fig. 6E). Inhibition of TRPA1 in the absence of Sema4C did not affect mechanical sensitivity (Supplementary Fig. 6E, F). Importantly, Sema4C-evoked mechanical hypersensitivity was strongly reduced in mice lacking TRPA1 (TRPA1^−/−^ mice) at 3–4 h post Sema4C injection, but only moderately so between 7–8 h (Fig. 6f). These differences between pharmacological and genetic manipulation of TRPA1 could arise from the basal mechanical pain defects found in TRPA1^−/−^ mice or from drug specificity issues.

### Sema4C-Plexin-B2-RhoA signaling modulates TRPA1 function

Based on the above results, we hypothesized that Sema4C-Plexin-B2 signaling via the RhoA-ROCK pathway sensitizes TRPA1 function.

**Fig. 7:**
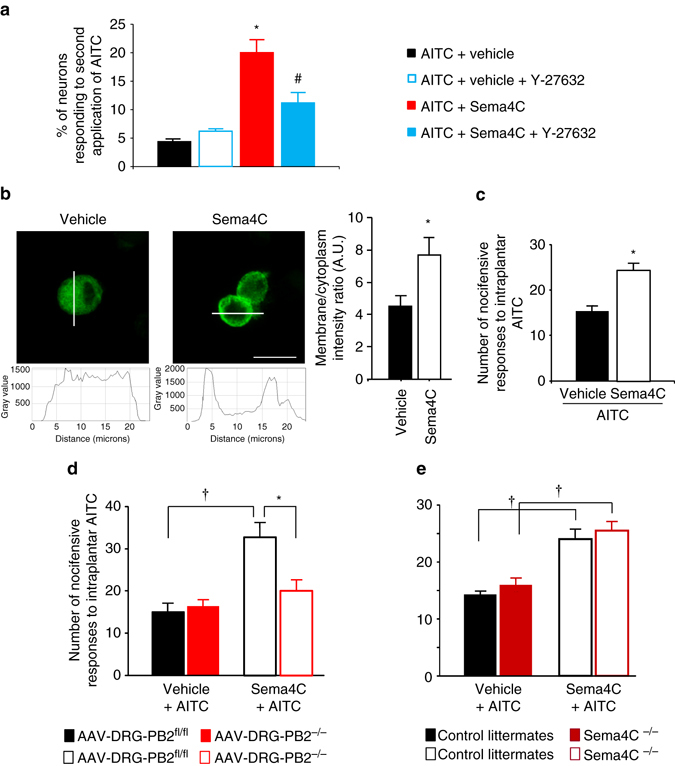
Modulation of TRPA1 channels by Sema4C/Plexin-B2/RhoA-ROCK signaling. **a** Modulation of calcium transients evoked in DRG sensory neurons by AITC (50 µM) by pretreatment with either vehicle or Sema4C (150 nM) in the presence or absence of ROCK inhibitor (50 μg/ml); *n* = 3 independent culture experiments. Note that AITC was applied together with Sema4C or vehicle treatment and again 30 min later alone - the responses elicited by a second application of AITC are shown in **a**. **b** Sema4C-induced changes in subcellular localization of myc-tagged TRPA1 in cultured DRG neurons. Shown are typical examples, line profiles on confocal images and quantification of fluorescence intensity at the cell membrane relative to cytoplasmic localization (*n* = 40 cells/group for Sema4C treatment and *n* = 43 for vehicle treatment). *Scale bar*, 25 µm. **c** Nocifensive responses to intraplantar application of Allyl isothiocyanate (AITC, 3.5 mM), a TRPA1 agonist, 3–4 h following intraplantar injection of vehicle or Sema4C (*n* = 5 mice/group for vehicle treatment, *n* = 8 for Sema4C treatment). **d**, **e** Nocifensive responses to intraplantar application of AITC following intraplantar injection of vehicle or Sema4C in mice lacking Plexin-B2 conditionally in adult DRG (AAV-DRG-PB2^−/−^) and their corresponding AAV-DRG-PB2^fl/fl^ controls (*n* = 8 mice/group), **d** or in mice lacking Sema4C (Sema4C^−/−^) and their wild-type littermates (*n* = 6 mice/group), **e**. ANOVA for random measures followed by Tukey’s test was performed in **a**, **d** and **e**, Student’s *t*-test was performed in **b** and **c**. In all panels, *P* < 0.05 indicated by * as compared to the corresponding control groups and by ^†^ as compared to basal and by ^#^ as compared to Sema4C-treated group. Error bars represent s.e.m

In Fura2-based calcium imaging experiments on cultured DRG neurons, the proportion of sensory neurons responding to the first application with AITC (~30%) was similar when neurons were pretreated with either PBS or Sema4C (Supplementary Fig. 6G). In vehicle-pretreated cultures, ~5% of neurons showed responsivity to a second application of AITC, as expected from previous studies^33^. In contrast, in Sema4C-treated cultures, nearly 20% neurons responded to a second application of AITC (Fig. 7a). Interestingly, this effect of Sema4C was abrogated when Y-27632, a ROCK1 inhibitor, was co-applied with Sema4C, indicating that this potentiating function of Sema4C is mediated via the RhoA-ROCK pathway (Fig. 7a). This suggests that Sema4C facilitates responsivity to TRPA1 agonists likely via enhancing the surface expression of TRPA1. Indeed, in DRG neurons transfected with myc-tagged TRPA1, activation of endogenously expressed Plexin-B2 via Sema4C led to enhanced localization of TRPA1 in the membrane of cultured DRG neuron somata (Fig. 7b). This indicates that activated Plexin-B2 increases membrane availability of TRPA1 in sensory neurons, which may underlie the potentiation of TRPA1-mediated functions, which we observed in calcium imaging experiments.

Sensitization of TRPA1 function can be addressed in vivo by studying behavioral responses to its ligand, namely AITC (3.5 mM concentration)^34^ and in vitro by analyzing calcium transients evoked in cultured DRG neurons by AITC^26^. Indeed, we observed that nocifensive behaviors evoked by intraplantar injection of AITC, such as licking and flicking of the injected hindpaw, were significantly potentiated when Sema4C was administered intraplantar 3 h before AITC treatment as compared to pretreatment with vehicle (Fig. 7c). We then tested the contribution of Plexin-B2 to the above effects. Whereas Sema4C treatment led to potentiation of AITC-induced nocifensive behaviors in control AAV-DRG-PB2^fl/fl^ mice, mice with adult-onset loss of Plexin-B2 in DRG neurons (AAV-DRG-PB2^−/−^) showed a complete lack of TRPA1 sensitization upon Sema4C treatment (Fig. 7d). Nocifensive behaviors induced by AITC alone were not affected in AAV-DRG-PB2^−/−^ mice, indicating that basal activity of TRPA1 is unaltered in the absence of Plexin-B2 (Fig. 7d). In contrast, treatment with exogenous Sema4C potentiated AITC-induced nocifensive behaviors in Sema4C^−/−^ mice to a similar extent as in wild-type mice, indicating that Plexin-B2-mediating signaling per se is intact in Sema4C^−/−^ mice (Fig. 7e).

### Role of PlexinB2-RhoA signaling in nociception in vivo

Because the above-described pharmacological analyses were based upon exogenous application of Sema4C, we found it important to delineate the role of RhoA signaling in vivo in the context of endogenous Plexin-B2 function in nociception. We have recently described a genetic strategy of specifically mutating Plexin-B2 domains wherein activation of specific downstream pathways is hindered. This is based on transgenic rescue of Plexin-B2 expression in global Plexin-B2 knockout mice (PB2^−/−^), brought about by using a bacterial artificial chromosome (BAC) containing the endogenous promoter elements of Plexin-B2 to express either wild-type Plexin-B2 or Plexin-B2 with mutations in specific domains^20^. Whereas PB2^−/−^ mice demonstrate embryonic lethality^1^, rescuing endogenous Plexin-B2 expression via the wild-type Plexin-B2 BAC fully restores viability^20^; referred to henceforth as PB2^−/−^; PB2^wt^ mice). Here, we observed that unlike SNS-PB2^−/−^ mice, PB2^−/−^; PB2^wt^ mice responded normally to nociceptive stimuli and were indistinguishable from wild-type mice (Supplementary Fig. 7A), indicating that Plexin-B2 expression was indeed rescued in sensory neurons.

**Fig. 8:**
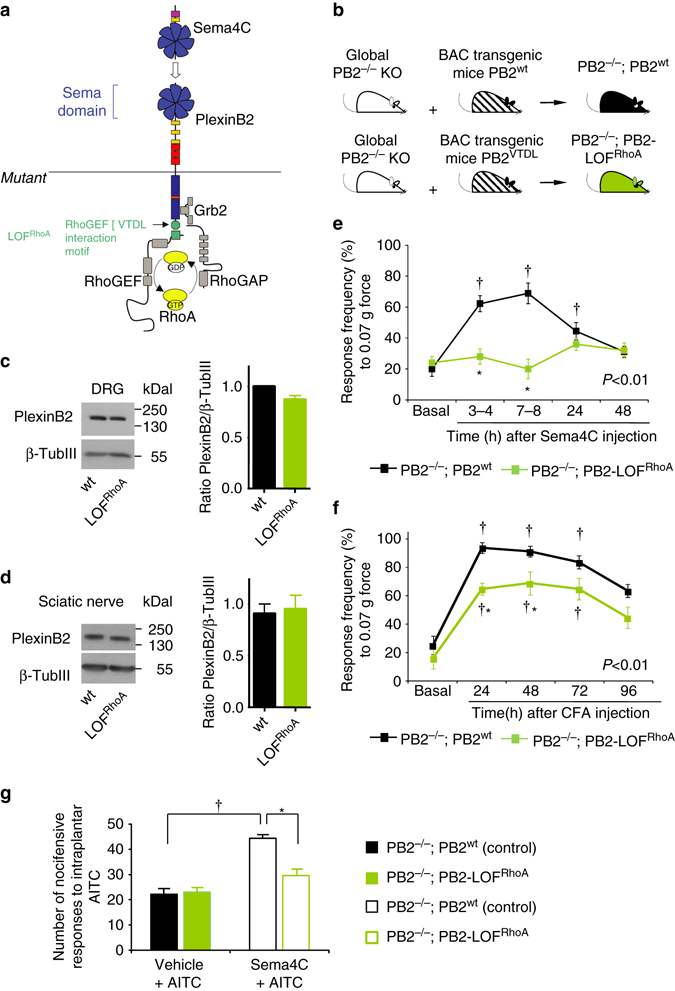
RhoA-ROCK signaling downstream of Plexin-B2 activation mediates Sema4C-induced mechanical hypersensitivity and contributes to inflammatory hypersensitivity in vivo. **a**, **b** Schematic representation of the allelic series of transgenic Plexin-B2 mice carrying specific mutations in the intracellular domain resulting in loss-of-function (LOF) of the RhoA activation pathway. **c**, **d** Examples (*left*) and densitometric quantifications (*right*) of western blot analysis of PlexinB2 signal in lysates of L3-L4 DRGs (**c**, *n* = 4 mice/group) or Sciatic nerve tissue (**d**, *n* = 3 mice/group) of transgenic Plexin-B2 mice carrying specific mutations in the intracellular domain resulting in LOF of the RhoA activation pathway (PB2^−/−^; PB2-LOF^RhoA^) and the control mice carrying wild-type (wt) Plexin-B2 (PB2^−/−^; PB2^wt^). Data are represented as fold changes of the ratio of PlexinB2 over loading control signal β-TubulinIII, *p* > 0.05. **e** Analysis of Sema4C-induced mechanical hypersensitivity to plantar application of 0.07 g von Frey force in PB2^−/−^; PB2-LOF^RhoA^ and control mice. **f** Analysis of inflammatory hypersensitivity, represented as response frequency to 0.07 g force before and at 24, 48, 72 and 96 h following hindpaw CFA injection. *N* = 5 mice/group for PB2^−/−^; PB2-LOF^RhoA^ mice and *n* = 8 for control mice in all experiments **e** and **f**. **g** Nocifensive responses to intraplantar application of AITC following intraplantar injection of vehicle or Sema4C in PB2^−/−^; PB2-LOF^RhoA^ and control PB2^−/−^; PB2^wt^ mice (*n* = 8 mice/group). Student *t*-test was performed in **c** and **d**, ANOVA for repeated **e**, **f** or random **g** measures followed by Tukey’s test was performed. In **e** and **f**, *P* values upon comparing entire curves with each other are represented. In all panels, *P* < 0.05 indicated by * as compared to the corresponding control groups and by ^†^ as compared to basal. Error bars represent s.e.m

Deletion of the C-terminal VTDL motif hinders RhoA signaling^6^; accordingly, we have recently described the generation of mice expressing a Plexin-B2 loss-of-function (LOF) mutant with respect to RhoA activation, which are viable and develop normally^20^; referred to henceforth as PB2^−/−^; PB2-LOF^RhoA^; Fig. 8a, b). In western blot analysis on DRG and on sciatic nerve segments isolated from the hindlimbs of PB2^−/−^; PB2-LOF^RhoA^ mice, we observed that the RhoA mutant of Plexin-B2 is indeed expressed and transported to the hindlimb and is comparable in magnitude to the expression of wild-type Plexin-B2 in control mice (Fig. 8c, d; full uncropped immunoblots are shown in Supplementary Fig. 8H, I). In behavioral experiments, we noted that the loss of RhoA activation via Plexin-B2 did not lead to significant deviations in basal sensitivity to mechanical stimuli or heat (Supplementary Fig. 7B, C), which was consistent with normal development of nociceptive fibers in PB2^−/−^; PB2-LOF^RhoA^ mice (Supplementary Fig. 7D). However, whereas PB2^−/−^; PB2^wt^ mice developed significant mechanical hypersensitivity upon intraplantar application of 1 ng Sema4C (Fig. 8e), PB2^−/−^; PB2-LOF^RhoA^ mice did not show any deviations from basal sensitivity upon treatment with Sema4C (Fig. 8e; Supplementary Fig. 7E). Thus, consistent with our results from pharmacological experiments described above (Fig. 6), Plexin-B2-mediated RhoA activation was observed to be obligatory for nociceptive sensitization by Sema4C.

Importantly, the magnitude of mechanical hypersensitivity evoked by paw inflammation was significantly attenuated in PB2^−/−^; PB2-LOF^RhoA^ mice and they showed a significantly shorter duration of mechanical hypersensitivity post CFA injection as compared to PB2^−/−^; PB2^wt^ mice (Fig. 8f; Supplementary Fig. 7F). In contrast, the development and maintenance of inflammatory thermal hyperalgesia was not hindered by deficits in Plexin-B2-mediated RhoA (Supplementary Fig. 7G). Importantly, Sema4C-induced potentiation of nocifensive responses evoked by intraplantar AITC was completely abrogated in PB2^−/−^; PB2-LOF^RhoA^ mice, thereby supporting the mechanistic link between Plexin-B2, RhoA-ROCK signaling and TRPA1 sensitization (Fig. 8g). Thus, pharmacological as well as genetic experiments point to a critical role for Plexin-B2-dependent RhoA-ROCK signaling in mechanical hypersensitivity post-inflammation.

## Discussion

This study reports a novel role for Plexin-B2-Sema4C signaling in peripheral sensory neurons in modulating inflammatory nociceptive hypersensitivity during adult life. Although SNS-PB2^−/−^ mice showed impairment in the basal ability to sense mechanical and thermal nociceptive stimuli, this emerged to be a consequence of developmental defects in sensory neurons, since bypassing developmental aberrations via adult-onset Plexin-B2 deletion in DRG neurons did not impact on basal nociception. This study does not have a developmental focus. Instead, we report that a developmentally important system is rekindled in adult life during a persistent pain state to promote both acute and long-lasting plasticity of sensory neurons, resulting in nociceptive hypersensitivity.

What is the identity and location of the ligand-receptor pair mediating nociceptive hypersensitivity? The potential involvement of other class IV semaphorins that have been shown to act on Plexin-B2 mostly in heterologous systems^2–4^ is not excluded. However, the observations that mice lacking Sema4C demonstrated a similar defect in development of mechanical hypersensitivity as Plexin-B2 mutants, and that exogenously administered Sema4C could recapitulate these sensory changes in a strictly Plexin-B2-dependent manner, indicate that Sema4C-Plexin-B2 constitute a key ligand-receptor pair operational in adult DRG neurons. Given that both the receptor and the ligand are broadly expressed in adult DRG neurons as well as in peripheral tissues, both autocrine and paracrine interactions between Plexin-B2 and Sema4C are possible, particularly in inflammatory states. Thus, the observed upregulation of both Plexin-B2 and Sema4C in DRG neurons and particularly, that of Sema4C in keratinocytes as well as cells of immune origin, such as macrophages and T cells, in the inflamed skin tissue, where Plexin-B2-expressing sensory neurons terminate, suggests that these ligand-receptor interactions are promoted in inflamed tissue. Interestingly, the differences observed between the developmental phenotypes in Plexin-B2 and Sema4C mutant mice indicate that semaphorin ligands other than Sema4C may activate Plexin-B2 in the course of development of the nociceptive circuitry, which is consistent with reports on other low-affinity ligands for Plexin-B2^1^.

Amongst the diverse families of plexins, most is known about the intracellular signaling cascades of B-type plexins, of which Plexin-B2 has been studied so far in the in vivo context. We and others have previously shown that a myriad of prominent branches of molecular signaling can be recruited by distinct domains of B-type plexins in cell lines and heterologous systems^5–10^; the picture emerging from recent in vivo studies is that particular developmental or pathophysiological functions of Plexin-B2 rely specifically upon particular pathways and there is a striking diversity of pathways underlying the different in vivo functions. Thus, we have recently observed that early embryonic developmental functions of Plexin-B2 in the nervous system, such as in the closure of the neural tube or cerebellar migration, are entirely mediated by the Ras GAP domain^20^. In contrast, the RhoA activation function of Plexin-B2 was found to be dispensable for neural development^20^.

Small RhoGTPases, such as RhoA, directly and profoundly regulate the actin cytoskeleton and have accordingly described to bring about morphological changes in neurons downstream of trophic factors and navigational cues during embryonic development^35^. However, they have not been associated with pain and sensitization of adult peripheral sensory neurons so far. How would RhoA/ROCK signaling mechanistically modulate the sensitivity of sensory neurons? Several scenarios are conceivable. One, given that actin rearrangement plays a role in regulating neurotransmitter release via movement of vesicles^36^, recruitment of RhoA-ROCK signaling in peripheral nociceptors may result in modulation of release of neurotransmitters and neuromodulators involved in sensitization. Alternatively, actin-mediated insertion of endocytotic vesicles may be regulated^37^. One mechanism for which we obtained experimental evidence was potentiation of TRPA1 function and membrane availability, both in vitro as measured via calcium imaging and membrane localization analyses, and in vivo as analyzed in terms of behavioral sensitivity to algogens acting via TRPA1. Because TRPA1-mediated basal calcium flux was not altered, Plexin-B2-mediated RhoA-ROCK signaling is unlikely to be altering the channel properties directly; rather our data indicate enhanced membrane availability of the channel in sensory neurons, as analyzed via membrane localization in sensory neurons as well as a previously validated in vivo assay^38^. It must be clarified, however that in mice lacking TRPA1, Sema4C-induced mechanical hypersensitivity was strongly attenuated at 3–4 h post Sema4C administration but only partially attenuated between 7 and 24 h post Sema4C treatment. This suggests that downstream mediators other than TRPA1 could also contribute to mechanical hypersensitivity induced by Sema4C. Moreover, TRPA1 is expressed in a sub-population of TRPV1-expressing sensory fibers and TRPV1-expressing fibers have been linked to thermal nociception rather than the mechanical modality of nociception^39^. Thus, the precise type of sensory neurons and afferents mediating Sema4C-induced sensitization and their link to TRPA1 needs further clarification.

Previous studies have implicated diverse RTKs, such as TrkA, TrkB, C-kit, VEGFR1, amongst others, in sensitization of peripheral nociceptive neurons, underlying the important pronociceptive contributions of trophic factors such as NGF, BDNF, VEGF, amongst others, in pathological pain states^27, 40, 41^, TrkA- and VEGFR1-dependent RTK signaling has been particularly shown to increase surface expression of the proton-, algogen- and heat-sensor, TRPV1^27, 42^. Although B-type plexins can activate Met, and Met signaling has been implicated in trophic development of sensory neurons^43^, pharmacological inhibition of Met did not affect Sema4C-Plexin-B2-mediated nociceptive sensitization. Given the close causal links between RTK activation, TRPV1 trafficking/sensitization and thermal hypersensitivity^27, 42^, this is also consistent with the observation that Plexin-B2 mutant mice only showed minor impairments in inflammatory thermal hyperalgesia. Thus, the nature and function of the effector systems modulated downstream of Plexin-B2 signaling may account for the stronger impact we observed on mechanical hypersensitivity as compared to thermal hypersensitivity despite the broad expression of Plexin-B2 across DRG neurons.

Taken together, our results demonstrate that Plexin-B2 plays non-redundant and distinct functional roles in the development of peripheral somatosensory neurons and their pathophysiological alterations in adult life, particularly in persistent inflammatory hypersensitivity, where it responds to Sema4C originating from non-neuronal cells in inflamed tissue. It is also plausible that this pathophysiological role of Plexin-B2 also extends to other types of chronic pain, such as cancer pain given the pronounced expression and induction of semaphorins in diverse types of cancers^15, 43, 44, 45^. Thus, these results may carry therapeutic relevance in the treatment of pathological pain.

## Methods

### Genetically modified mice

All animal use procedures were in accordance with ethical guidelines imposed by the local governing body (Regierungspräsidium Karlsruhe, Germany). Mice used were 8–20 weeks of age.

The *plxnb1*, *plxnb2* and *sema4c* genes were mutated by targeted trapping resulting in functional null alleles and expression of β-galactosidase (LacZ) under the control of the respective endogenous promoters^3, 16, 46^. Mice lacking Plexin-B2 in sensory neurons were generated by crossing mice carrying a conditional allele for Plexin-B2 (PB2^fl/fl^) gene^1^ with SNS-Cre mice^23^ to obtain SNS-Cre^+^, PB2^−/−^ mice (referred to as SNS-PB2^−/−^ in the manuscript). Mice genetically lacking TRPA1 and their corresponding wild-type mice were obtained from the Jackson Laboratory.

Sema4C-LacZ reporter mice (heterozygous) and mice lacking Sema4C (homozgous Sema4C^LacZ/LacZ^ mice) as well as their corresponding wild-type littermates has been described previously^3^.

The generation of mice expressing triple-myc-tagged wild-type Plexin-B2 or triple-myc-tagged Plexin-B2(ΔVTDL) has been described elsewhere^20^.

All mice were from the C57Bl6 background. Age-matched mice (between 8 and 20 weeks old) of both sexes where used throughout all experiments, with the exception of experiments involving mice with a loss of RhoA function, where only males were used. Mice were housed in the central facility of Heidelberg University in groups of 2–4 mice/group on a 12 h light-dark cycle with constant room temperature.

### AAV-Cre injection into the DRGs of Plexin-B2^fl/fl^ mice

We used adeno-associated viruses (AAV) expressing iCre under the synapsin promoter. The AAV serotypes used yield broad and neuron-specific expression and lead to a neuron-specific expression of CRE recombinase^25, 26^. Injections of AAV virions into the DRGs in vivo were performed as described in details previously^25^. Briefly, Plexin-B2^fl/fl^ mice at 8–10 weeks of age were anesthetized with a mixture of dormicum/domitor/fentanyl (3:8:2, 1 µl/g i.p.). 500 nl of a 2:1 mixture of AAV viral stocks with 20% Mannitol containing 1.2 × 10^7^ virions were injected into the right side of L3-L4 DRGs (total of 2 injections per mouse) at a flow rate of 8.3 nl/min using a glass pipette and a microprocessor-controlled minipump (World Precision Instruments, WPI Germany). Mice were allowed to recover for at least 3 weeks before commencing behavioral analysis. A detailed characterization of the rate of transduction in DRG neurons, neuron-specificity and lack of toxicity is given in our previous study^26^. After the behavioral testing every mouse was sacrificed and monitored for the viral spread by extracting the L3 and L4 DRGs and then assessing the DRGs by GFP fluorescence. Mice not showing broad GFP expression in both L3, L4 DRGs injected were excluded from the study (less than 20%).

### Analysis of β-galactosidase activity

Mice were transcardially perfused with cold phosphate-buffered saline (PBS) and with 0.5% cold paraformaldehyde (PFA). Tissues were post fixed for 6 h in 0.5% PFA in PBS at 4 °C followed by an overnight incubation in 30% sucrose in PBS at 4 °C. Tissues were frozen and cryosectioned at 25 µm (skin) and at 16 µm (DRG, embryo). Sections were stained in staining solution (0.1 M PBS pH 7.3, 2 mM MgCl_2_, 5 mM EGTA, 0.01% sodium deoxycholate, 0.02% NP-40, 10 mM potassium ferricyanide, 10 mM potassium ferrocyanide, 0.5 mg/ml X-gal) at 37 °C in the dark. Corresponding wild-type tissue was routinely included and entirely failed to yield signals.

### Immunohistochemistry on paw punches, DRG and spinal cord

Mice were transcardially perfused with cold PBS and with 4% cold PFA and plantar punch biopsies of the hindpaw skin, L3-L4 DRGs, or L3-L4 spinal cords were extracted, cryopreserved in 30% sucrose and cryosectioned at 25 µm (skin), 16 µm (DRG) and 20 µm (spinal cord). Paw sections were stained with anti-CGRP antibody (1:800; 24112, Immunostar), anti-PGP9.5 antibody (1:500, RA96101, Ultraclone), anti-Gr-1 antibody (1:500, 553125, BD Biosciences), anti-CD3 antibody (1:100, 555273, BD Biosciences) or anti-Sema4C antibody (rabbit 1:25, CSB-PA787154, CusAb-Cusabio). DRGs were stained with anti-β-galactosidase antibodies (rabbit 1:700; MP Biomedicals, 559762 and chicken 1:800; Abcam, ab9361), anti-PlexinB2 (rabbit 1:25, CSB-PA347467, CusAb-Cusabio), anti-CGRP antibody (1:2000; 24112, Immunostar), biotinylated Isolectin B_4_ (1:200; B-1205, Vector), and anti-NF200 antibody (1:300, N4142, Sigma).

Spinal cord sections were stained with anti-CGRP antibody (1:2000; 24112, Immunostar) and with biotinylated Isolectin B_4_ (1:200; B-1205, Vector). All the stainings were completed by using standard protocols^25^ with the exception of CD3 staining, and analyzed by using a confocal laser-scanning microscope (Leica TCS SP2 AOBS or Leica TCS SP8 AOBS). For single or double immunostaining of T cells, paw sections where fixed with 100% ethanol, washed and incubated with 100% acetone. After incubation in blocking solution containing 5% normal horse serum, 0.2% Tween-20 and 0.3% Triton X-100, paw sections were than incubated overnight at 4 °C with a primary anti-CD3 antibody (1:100, 555273, BD Biosciences) diluted in PBS solution containing 5% BSA.

### Western blotting

L3-L4 mouse DRG or mouse paw tissues were homogenized in ice-cold hypotonic buffer (25 mM Tris pH 7.4, 5 mM EDTA, 1 mM DTT) using a dounce homogenizer^18^. An equal volume of 2× ice-cold radioimmunoprecipitation buffer (600 mM NaCl, 100 mM Tris pH 7.4, 10 mM EDTA, 2% Triton X-100, 0.2% SDS, 1% sodium deoxycholate) was added to the tissues. The lysates were rotated for 30 min at 4 °C, centrifuged for 20 min at 4 °C, and supernatants were used for western blotting according to standard protocol.

Western blots were performed with the following antibodies: anti-Sema4C (sheep 1:200, PA5-47812, ThermoFisher Scientific), anti-PlexinB2 (sheep 1:700, PA5-47880, ThermoFisher Scientific), anti-Phospho Met (Phospho-Met (Tyr1234/1235) (D26) XP® Rabbit mAb 1:1000, # 3077, Cell Signaling Technology (CST)) Antibodies recognizing β-tubulinIII (rabbit 1:6000, T2200, Sigma Aldrich) or GAPDH (goat (V-18) 1: 500, sc-20357, Santa Cruz) were used as loading controls. Membranes were incubated with anti-rabbit (1:6000), or anti-sheep (1:4000) or anti-goat (1:4000) HRP-conjugated antibodies and developed using Amersham™ ECL™ (GE Healthcare) and Hyperfilm MP (Amersham). At least five samples from independent experiments were analyzed by densitometry using Fiji (ImageJ) and corrected for loading by normalization to corresponding bands for β-tubulin or GAPDH.

In some experiments, recombinant mouse HGF (50 ng; R&D System, 2207-HG-025/CF), in presence or absence of an inhibitor of Met signaling (PHA 665752, 3.3 μg, Tocris), was injected unilaterally intraplantar in the hindpaw and changes in levels of P-Met were detected, as described above.

Supplementary Fig. 8 shows the full and uncropped blot pictures related to all cropped western blot examples that were included in the main and Supplementary Figures.

### RNA extraction and qRT-PCR

Tissue was harvested from the L3-L5 DRGs or cultured DRGs, shock-frozen on dry ice and total RNA was extracted using the Trizol method (Invitrogen) and purification steps using Turbo DNAse (Ambion) and RNAse out (Invitrogen) were employed as per manufacturer´s instructions. For the generation of first strand cDNA, 1 µg of total RNA was reverse transcribed by extension of oligo(dT)20 primers using SuperScript III reverse transcriptase (Invitrogen) according to the manufacturer’s instructions. In some samples, reverse transcriptase was omitted to serve as a control. qPCR reactions were performed on cDNA samples using theTaqMan Gene Expression Assay (Invitrogen) including predesigned primers for PlexinB2 and GAPDH (Invitrogen). The reactions were performed using a LightCycler 96 Real Time PCR System (Roche) and the data analyzed using the corresponding software. The expression level of the target mRNA was normalized to expression of *Gapdh* RNA. qRT-PCR assay was performed in triplicates.

### Nociceptive tests

All animal use procedures were in accordance with ethical guidelines imposed by the local governing body (Regierungspräsidium Karlsruhe, Germany). All behavioral measurements were done in awake, unrestrained, age-matched mice (between 8 and 14 weeks old) of both sexes, and the mice of various genotypes were randomly assigned to treatment groups by the same investigator who was blinded to the identity of the groups in all behavioral tests. Mice chosen for behavioral testing were kept in individual cages on a 12 h light-dark cycle with constant room temperature. Behavioral testing was performed during the light (day) phase.

Mice were habituated to the experimental setup for the von Frey test and the Hargreaves test several times before the analysis and 30–60 min immediately before each experiment. Mechanical sensitivity was tested by using von Frey monofilaments of different forces (Ugo Basile) ranging from 0.04 to 1.4 g. Paw withdrawal responses to five applications of each filament were tested^24^. Thermal sensitivity was examined with the Hargreaves test and with the Hot/Cold Plate test. For the Hargreaves test infrared heat was applied with a Hargreaves apparatus (Ugo Basile) to the plantar surface of the hindpaw and the latency to the withdrawal of the paw (thermal latency) was measured, using 15 s as the cutoff latency^24^. The hot plate test was done at 50 °C (25 s cutoff latency) with a Hot/Cold Plate (Bioseb, Vitrolles, France). The latency until the first withdrawal response of the hindpaw was recorded. The mice were removed immediately after the response. The intraplantar AITC test and capsaicin test was performed as described^25, 47^. Briefly, 3.5 mM Allyl isothiocyanate (AITC, Sigma-Aldrich, A33205) diluted in mineral oil in and 0.03% capsaicin (Tocris, 0642) diluted in PBS were injected intraplantar. The number of nocifensive responses including lifting, licking or flinching was quantitated.

In some experiments, recombinant Sema4C (Cusabo, CSB-BP723720MO) was injected unilaterally intraplantar in the hindpaw and changes in mechanical sensitivity were recorded, as described above.

Furthermore, the following pharmacological inhibitors were employed in behavioral experiments: PHA 665752, an inhibitor of Met signaling (3.3 μg, Tocris), Y-27632, a ROCK 1/2 inhibitor (2 ng, Calbiochem-Merck) and AP-18, an inhibitor of TRPA1 (4.2 µg, Sigma).

### Rotarod

Motor coordination and balance control of the SNS-PB2^−/−^ mice and their corresponding PB2^fl/fl^ controls was tested using a rotarod test (TSE RotaRod System). The animals were trained over four consecutive days and tested on day 5 with the following two phase protocol. In phase 1, the mice were performing at the speed of 1 r.p.m. for 30 s. Next, in phase 2 the mice were running with acceleration from 1 to 50 r.p.m. over a 240 s period. Each day, the mice spent 1 h in the testing room prior to the test and performed three runs with 20 min breaks in between. The latency values were measured.

### Mouse models of inflammatory pain

For induction of paw inflammation, 20 µl CFA (Sigma-Aldrich, F5881) was injected unilaterally under isoflurane anesthesia into the intraplantar surface of the hindpaw^34, 48^.

### Extent of inflammation

Mice were transcardially perfused with 4% PFA in PBS under deep anesthesia. Paws were dissected and postfixed for 24h. Paw biopsy punches were cryosectioned and DAB staining with αGr-1 antibody was performed on cryosections (20 µm) paw sections. The inflammatory area was evaluated in microscopic images. The number of αGr-1-positive cells was calculated as ratio of the number over the measured area.

### DRG neuronal cultures

Eight weeks old mice were killed in CO_2_ and the DRGs were quickly collected in ice-cold PBS after removing axons and meningeal tissue and digested for 30 min at 37 °C in Trypsin, collagenase and DNAse. Neurons were separated from non-neuronal cells using a discontinuous Percoll gradient, than plated the on poly-L-lysine coated coverslips. The DRGs were cultured in F12 medium plus 10% normal serum in presence of a mixture of growth factors (BDNF 10 ng/ml, NGF 10 ng/ml, GDNF 5 ng/ml, NT3 5 ng/ml, all from Sigma-Aldrich) and a mitotic inhibitor AraC (5 µM, Sigma-Aldrich) for 3.5–4 days before treatment.

### Stimulation of cultured DRG neurons with Sema4C

3.5–4-day-old DRG neuronal cultures were starved by replacing the culture medium with starving medium containing no fetal bovine serum and growth factors for 1 h. Starving medium was replaced with culture medium containing 0.5% fetal bovine serum, no growth factors and containing 150 nM recombinant Sema4C for 5,10 and 15 min.

### Transfection cultured DRG neurons and immunofluorescence

Mice at 4–6 weeks of age were killed with CO_2_ and DRGs were quickly collected in cold PBS after removing axons and meningeal tissue and digested for 30 min at 37 °C in trypsin, collagenase and DNAse. Neurons were plated on poly-L-lysine coated coverslip and cultured in Neurobasal medium plus B27 and N2 supplement (Gibco) without adding growth factors or mitotic inhibitor. At 18–20 h after plating, cultured DRG neurons were transfected using the calcium phosphate method with the following plasmids: ORF of mouse TRPA1 C- terminally myc-tagged (a kind gift from Manuela Schmidt, Max Planck Institute für Experimentelle Medizin, Göttingen), or a control vector (Origene Inc.).

At 24 h after transfection, the cells were treated with Sema4C (see above) for 15 min, washed and fixed in 4% PFA for 20 min at room temperature. Immunofluorescence analysis was performed using an anti-myc antibody (mouse (9E10) 1:1000, sc-40 Santa Cruz) anti-β-tubulin III (rabbit 1: 1000; T2200, Sigma) and secondary antibodies conjugated with Alexa488 or Alexa 594 (Molecular Probes, Invitrogen) using standard protocols. The relative levels of TRPA1 expression at the cell membrane vs. cytoplasm were analyzed using the line profile tool in confocal microscopy (Leica TCS AOBS), or Fiji software.

### Rho-A activation assay

RhoA activity was determined by using a luminescence-based G-LISA™ RhoA activation assay kit (Kit # BK121, Cytoskeleton, Inc., Denver, CO) according to the manufacturer’s instructions. Briefly, stimulated DRG neuronal cultures were washed with ice-cold 1× PBS to get rid of any serum protein contaminants and total proteins were harvested by incubating with the cell lysis buffer containing protease inhibitors. Cell lysates were clarified of debris by centrifuging at 4 °C, 13000 RPM for 1 min. The protein concentration was determined according to manufacturer’s instructions using Precision Red™ Advanced Protein Assay Reagent provided in the kit and cell extracts were equalized to a protein concentration of 1.5 mg/ml for assay. Luminescence was detected according to the manufacturer’s recommendations after incubating for 45 min at room temperature with the primary anti-RhoA antibody supplied with the kit. Luminescence intensity was measured at 490 nm in Luminoskan™ Ascent Microplate Luminometer (5300173, Thermo Scientific, USA.) Cell lysis buffer and constituently active RhoA provided with the kit were used as negative and positive controls for the assay respectively.

### Calcium imaging

Wild-type mice (4–6 weeks old) were killed by inhalation of CO_2_. Bilateral spinal ganglia were removed in DMEM solution. DRG neurons were dissociated and plated on coverslips precoated with poly-lysine. At 24 h after culture, neurons were loaded with Fura-2 (10 μM) for 45 min. During 30 min of de-esterification period, cells were stained with IB4-FITC (1:200 stock 1 mg/ml). After a quick wash with mouse ringer solution calcium signals were recorded at a rate of 1 Hz using an upright fluorescence Olympus BX51WI microscope (Olympus, Japan) with 16X objective, a Sensicam charge-coupled device camera (PCO) and TILLvisION (T.I.L.L. Photonics). The TRPA1 agonist AITC (100 µM) was applied by bath application for 2 s followed by 10 min washing and 15 min incubation with Sema4C (150 nM) or vehicle (PBS). A quick wash to wash-out Sema4C was allowed prior a second application of agonist as described above. In some experiments at each step Rock 1–2 inhibitor Y-27632 (50 µg/ml = 147 µM) was applied. F340/380 ratios were calculated as described previously^49^. During data analysis, cells which were washed away during the procedure or which showed lack of a response to the second application of agonist or a > 10 % deviation in baseline ratios were ruled out from the data set.

### Statistics

All data are expressed as mean ± s.e.m. While comparing two groups with each other for a single parameter and single time point, Student´s *t*-test was employed. In experiments comparing multiple groups or multiple time points, analysis of variance (ANOVA) for random measures or repeated measures (in longitudinal experiments) was employed and *post-hoc* Tukey’s test for multiple comparisons was performed to determine statistically significant differences. All the statistical tests are two-sided unless otherwise specified in the figure legend. *P* less than or equal to 0.05 was considered significant. *P* values for each experiment are provided in the figure legends.

### Data availability

The authors declare that all data supporting the findings of this study are available from the corresponding author upon request.

## Electronic supplementary material

Supplementary Information
