# Supplementary material for: Semaphorin 4C Plexin-B2 signaling in peripheral sensory neurons is pronociceptive in a model of inflammatory pain
Source: Nat Commun. 2017 Aug 2;8:176. doi: 10.1038/s41467-017-00341-w (PMC5539317; doi:10.1038/s41467-017-00341-w)
Supplement: Supplementary file 1 — Supplementary Information [file 41467_2017_341_MOESM1_ESM.pdf]

File name: Supplementary Information
Description: Supplementary Figures

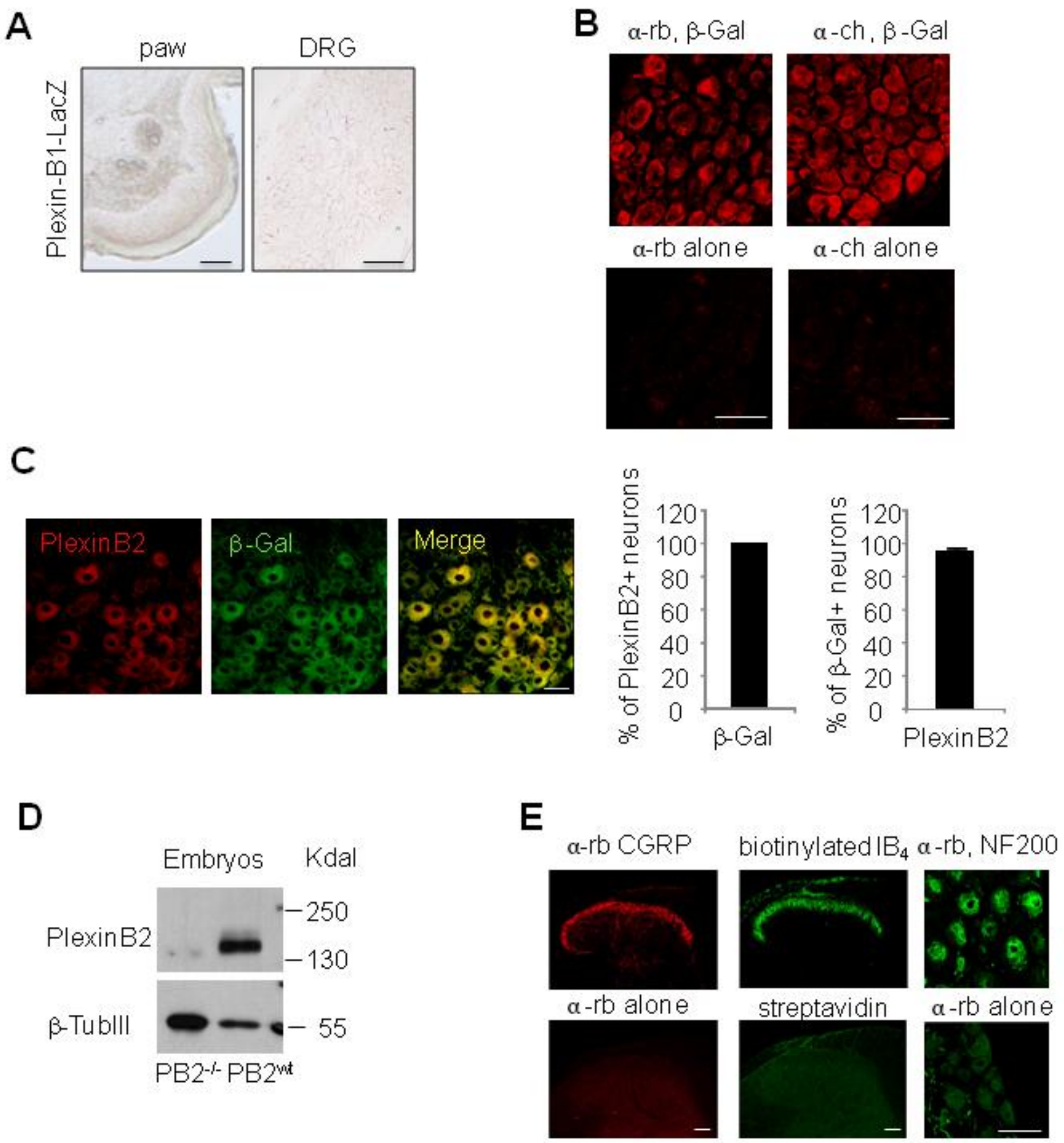

Supplementary Figure 1. Analysis of Plexin-B2 mutant mice and negative controls for immunofluorescence

(**A**) LacZ staining for analysing expression of Plexin-B1 in adult paw and DRGs by employing Plexin-B1-LacZ$^{+/-}$ indicator mice. Scale bars represent 100 µm. (B) Immunofluorescence analysis of expression of β-Galactosidase (β-Gal) in mouse lumbar DRG sections detected with the antibodies used in this study. Negative controls were run in parallel without primary antibodies. Scale bars represent 50 µm.

(C) Evidence for PlexinB2 coexpression with β-Gal in DRGs of Plexin-B2-LacZ$^{+/-}$ mice upon costaining with anti β-Gal andanti-PlexinB2 antibodies. Scale bar indicates 25 µm. (D) Typical example of Western blot analysis for Plexin-B2 showing transgenic rescue of Plexin-B2 expression in global Plexin-B2 knockout mice (PB2$^{-/-}$), brought about by using a bacterial artificial chromosome (BAC) containing the endogenous promoter elements of Plexin-B2 to express wild-type Plexin-B2. The lack of signal in PB2$^{-/-}$ mice is validation of specificity of the anti-Plexin-B2 antibody. (E) Negative controls for immunofluorescence staining in the absence of the respective primary antibodies. Scale bars indicate 75 µm.

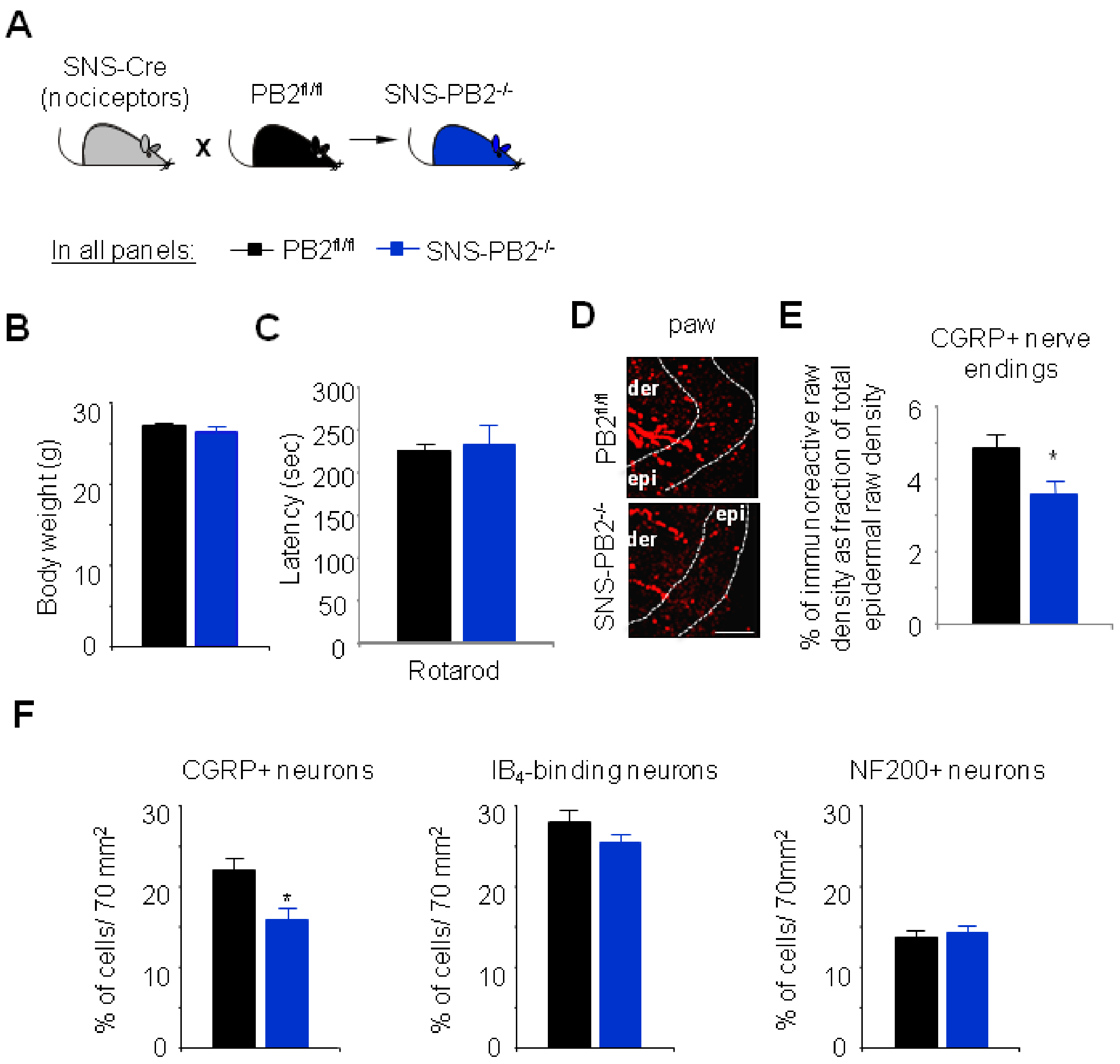

Supplementary Figure 2. Analysis of SNS-Plexin-B2 mutant mice

(A) Schematic representation of generation of mice conditionally lacking *plxnb2* specifically in nociceptive neurons (SNS-PB2$^{-/-}$). (B) Analysis of body weight of adult SNS-PB2$^{-/-}$ and their PB2$^{fl/fl}$ littermates (n = 4 for SNS-PB2$^{-/-}$ mice and n = 5 for PB2$^{fl/fl}$ mice). p>0.05 Student's t-test) (C) Analysis of motor coordination and balance control of the SNS-PB2$^{-/-}$ mice and their corresponding PB2$^{fl/fl}$ controls was tested using a rotarod test (TSE RotaRod System). n = 4 mice/group

(D, E) Typical examples (panel D) and quantitative summary (panel E) of the density of peptidergic nociceptor endings in the hindpaw plantar skin. Scale bar represents

50 µm (n = 18-22 sections/group taken from at least 3 different mice). (F) Quantitative analysis of the distribution of DRG neuronal sub-types in SNS-PB2$^{-/-}$ mice and their corresponding PB2$^{fl/fl}$ controls via immunolabeling with specific markers (n = 10-20 sections/group taken from at least 3 different mice).

In all panels Student's t-test was performed, $P<0.05$ indicated by * as compared to the corresponding control groups. Error bars represent S.E.M.

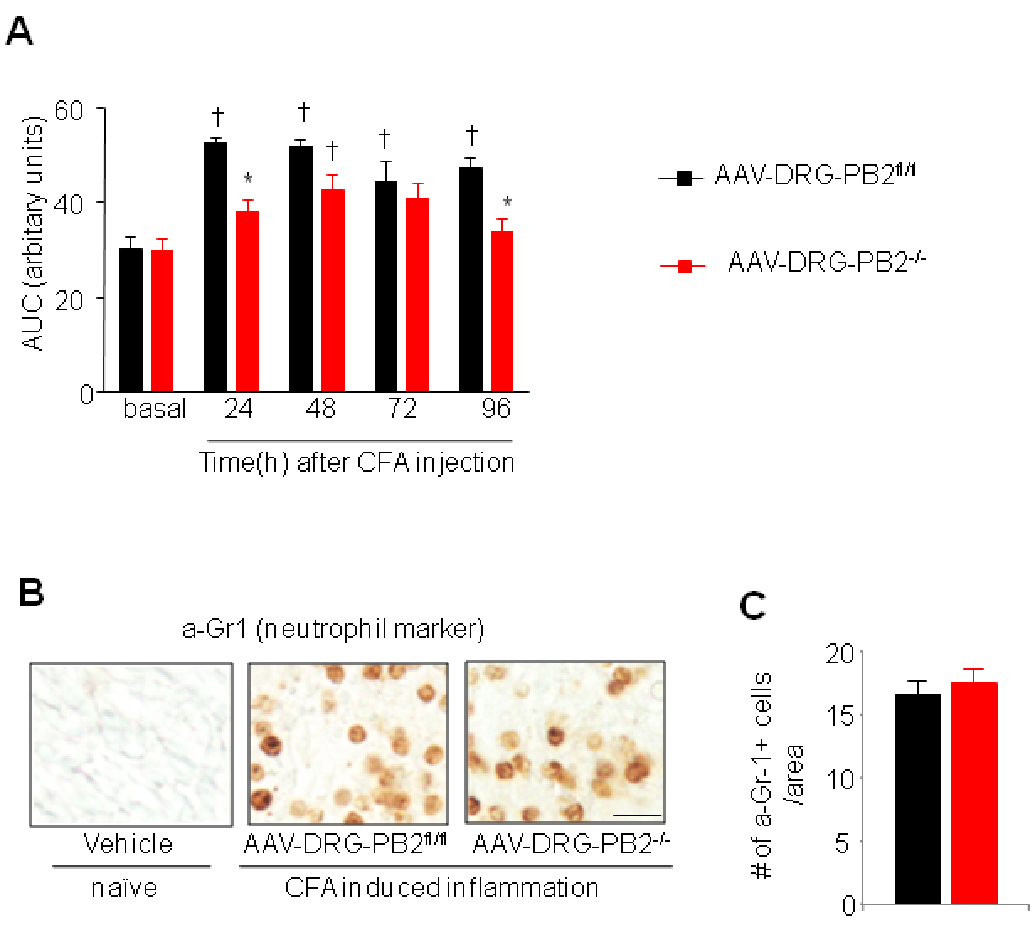

Supplementary Figure 3. Analysis of paw inflammation in AAV-DRG-PB2$^{-/-}$ mice

(A) Analysis of the magnitude and time-course of mechanical hypersensitivity induced by CFA-induced paw inflammation in AAV-DRG-PB2$^{-/-}$ mice and their corresponding AAV-DRG-PB2$^{fl/fl}$ controls (n = 7 mice/group in both cases).

(B, C) Typical examples (B) and quantitative summary of paw inflammation (C**)** which is indicated by infiltration of neutrophils (analysed via anti-Gr1 immunoreactivity) in the plantar skin (n = 3 mice/group). Scale bar indicates 20µm.

Student's t-test (panel C) and ANOVA for repeated measures (panel A) followed by Tukey's test were employed; $P<0.05$ indicated by * as compared to control mice and by † as compared to basal values within a group. Error bars represent standard error of the mean.

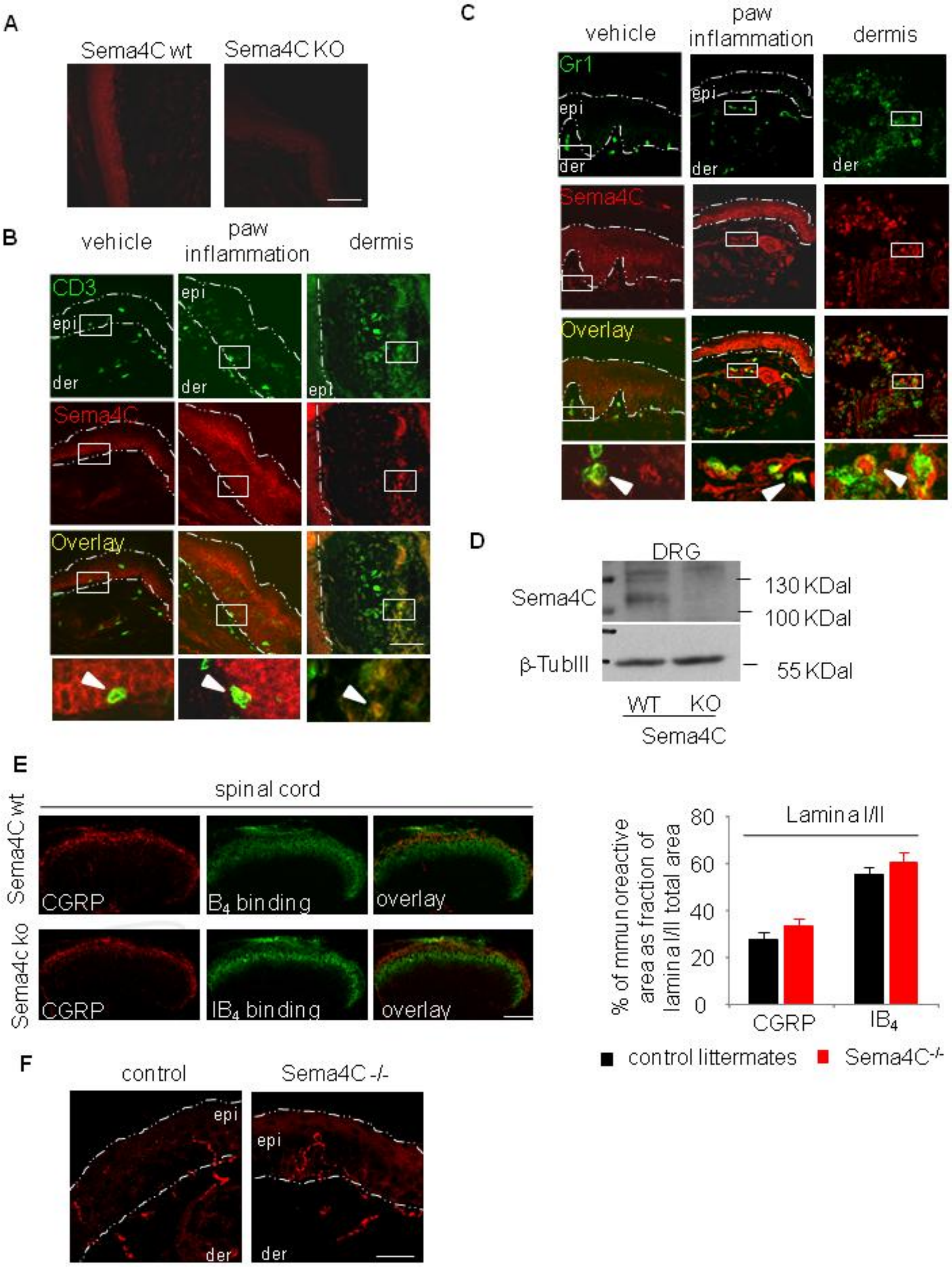
A
Sema4C wt
Sema4C KO
B
vehicle
paw inflammation
dermis
CD3
epi
der
Sema4C
Overlay
C
Gr1
D
DRG
Sema4C
130 KDal
100 KDal
β-TubIII
55 KDal
WT
KO
Sema4C
E
spinal cord
Sema4C wt
Sema4c ko
CGRP
B4 binding
IB4 binding
overlay
Lamina I/II
% of immunoreactive area as fraction of lamina I/II total area
80
60
40
20
0
CGRP
IB4
control littermates
Sema4C-/-
F
control
Sema4C -/-

Supplementary Figure 4. Analysis of Sema4C knockout (Sema4C$^{-/-}$) mice.

(A) Anti-Sema4C antibody yields significant staining on paw sections from wild-type mice but only background staining on paw sections from Sema4C$^{-/-}$ mice. Scale bar represent 50 µm. (B, C) Double immunocytochemistry of anti-Sema4C antibody together with antibodies against immune cells (anti-CD3 for T-cells (B) or anti-GR-1 for macrophages (C)) showing effects of CFA-treatment on Sema4C expression in paw tissue. Partial colocalization of Sema4C signal in dermis is shown at higher resolution in panels on the bottom with arrowheads. Scale bar represent 50 µm.

(D) Validation of the specificity of anti-Sema4C antibody in Western blotting. Anti-Sema4C antibody recognizes Sema4C expressed in DRG lysates from wildtype mice, but not in DRGs of mice genetically lacking Sema4C (Sema4C$^{-/-}$ mice).

(E) Typical examples and quantitative summary of immunohistochemical determination of the spinal targeting of CGRP-positive and IB_4-binding nociceptors (n= 14-18 sections/group taken from at least 3 different mice). Scale bar indicates 100 µm. (F) Typical examples of the density of peptidergic putative nociceptor endings in the hindpaw plantar skin in Sema4C $^{-/-}$ mice and littermate controls . Scale bar represents 50 µm.

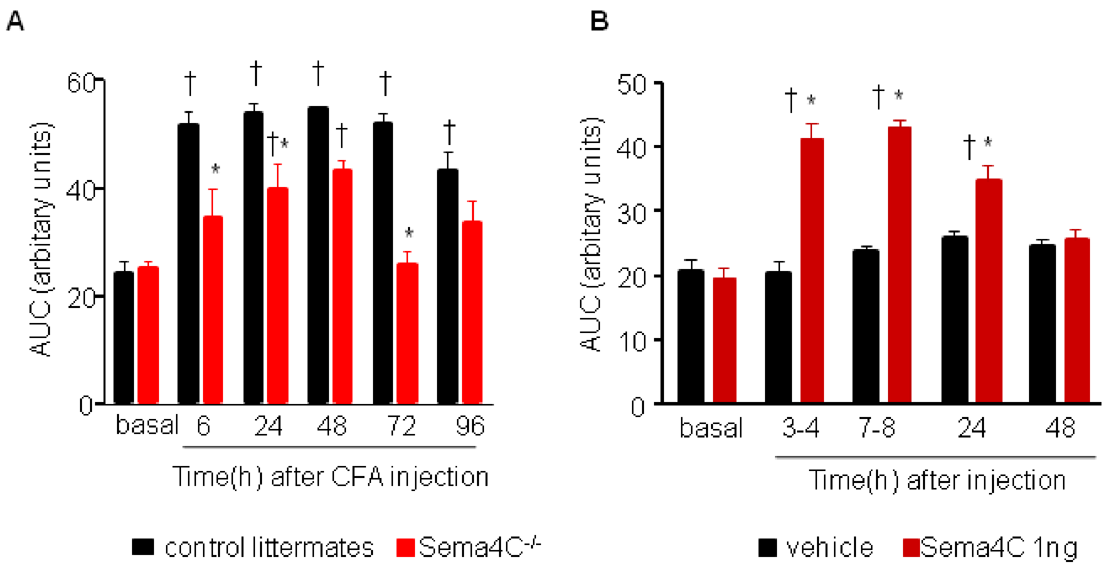

Supplementary Figure 5. Mechanical hypersensitivity in Sema4C$^{-/-}$ mice.

(A) Analysis of inflammatory mechanical hypersensitivity following hindpaw CFA injection in mice lacking Sema4C (Sema4C$^{-/-}$) and their wildtype littermates. Integral of responses to all graded mechanical von Frey stimuli (area under the curve of the stimulus intensity-response frequency curve) in mice before and at 6, 24, 48, 72, 96 hours following CFA injection is shown. (B) Time-course analyses of the effect on mechanical sensitivity of a single intraplantar dose of 1 ng Sema4C injection in mice lacking Sema4C (Sema4C$^{-/-}$) and their wildtype littermates. The integral responses to all graded von Frey filaments is shown. n = 7 mice/group for Sema4C -/- mice and n = 5 for wildtype littermates. Two-way ANOVA for repeated measures followed by Tukey's test was performed

In all panels, P<0.05 indicated by * as compared to the corresponding control groups and by † as compared to basal. Error bars represent S.E.M.

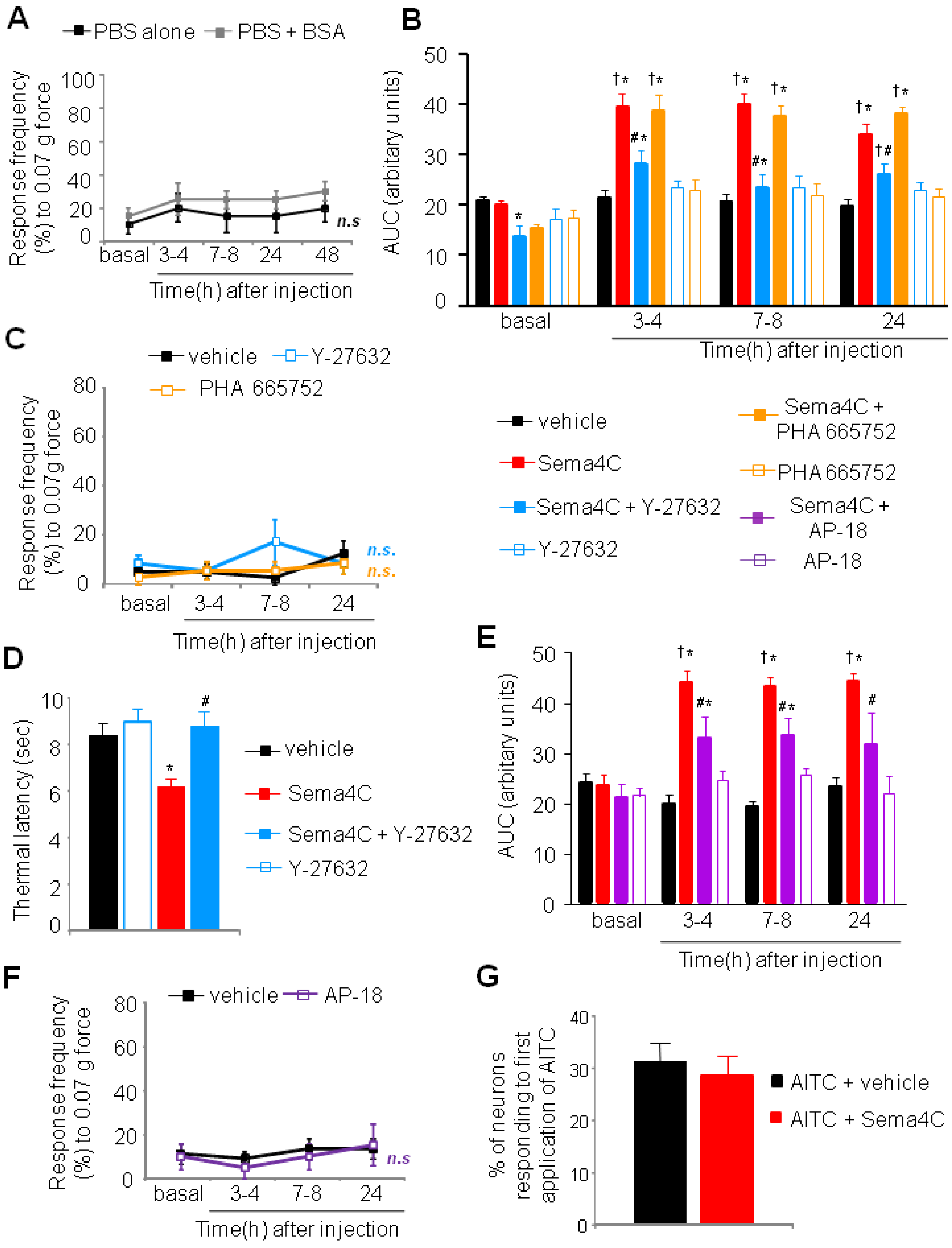
A
PBS alone
PBS + BSA
Response frequency (%) to 0.07 g force
100
80
60
40
20
0
basal
3-4
7-8
24
48
n.s
Time(h) after injection
B
AUC (arbitary units)
50
40
30
20
10
0
basal
3-4
7-8
24
Time(h) after injection
C
vehicle
Y-27632
PHA 665752
Response frequency (%) to 0.07g force
80
60
40
20
0
n.s.
n.s.
basal
3-4
7-8
24
Time(h) after injection
vehicle
Sema4C
Sema4C + Y-27632
Y-27632
Sema4C + PHA 665752
PHA 665752
Sema4C + AP-18
AP-18
D
Thermal latency (sec)
10
8
6
4
2
0
vehicle
Sema4C
Sema4C + Y-27632
Y-27632
E
AUC (arbitary units)
50
40
30
20
10
0
basal
3-4
7-8
24
Time(h) after injection
F
vehicle
AP-18
Response frequency (%) to 0.07 g force
80
60
40
20
0
n.s
basal
3-4
7-8
24
Time(h) after injection
G
% of neurons responding to first application of AITC
40
30
20
10
0
AITC + vehicle
AITC + Sema4C

Supplementary Figure 6. Analysis of blockade of diverse pathways on Sema4C-mediated hypersensitivity. (A) Paw withdrawal frequency in response to 0.07 g of von Frey filament application following intraplantar injection of PBS alone or PBS + bovine serum albumin (BSA) as an unspecific protein (n = 4 mice). (B) Area under the curve (AUC) before and 3-4, 7-8 and 24 hours following Sema4C, Sema4C+ROCK inhibitor (Y-27632), Sema4C+Met inhibitor (PHA 665752) or administration of the respective inhibitors alone alone (n = 7mice/group). (C) Frequency of paw withdrawal in response to 0.07 g of von Frey filament application following a single unilateral injection of Y-27632, PHA 665752 and vehicle alone in the absence of Sema4C (n = 7 mice/group). (D) Sema4C-induced thermal sensitivity 3-4h following a single intraplantar Sema4C injection in the presence or absence of Y-27632 (n=5 mice/group for Y-27632 and Y-27632+ Sema4C treatment, n = 7 for vehicle and Sema4C treatment). (E) Area under the curve before and 3-4, 7-8 and 24 hours following vehicle (n = 9 mice/group), Sema4C (n = 9 mice/group), Sema4C+TrpA1 inhibitor (AP-18) (n = 4 mice/group) or administration of the inhibitor alone alone (n = 4 mice/group). (F) Paw withdrawal frequency in response to 0.07 g force via von Frey hair application following intraplantar injection of AP-18 (n = 4) and vehicle (n = 9). (G) Proportion of isolated DRG neurons responding to first application of AITC (50 µM) in the presence of vehicle- or Sema4C, n=4 (Sema4C treatment) and 7 (Vehicle treatment) independent experiments. Student´s t-test (in panel G), ANOVA for random measures (in panel D) or ANOVA for repeated measures (panels A, B, C, E, F) followed by Tukey´s test were employed. Statistical significances (P<0.05) are indicated by * as compared to control group, by † as compared to basal values within a group and by # as compared to Sema4C treated group. Error bars, SEM.

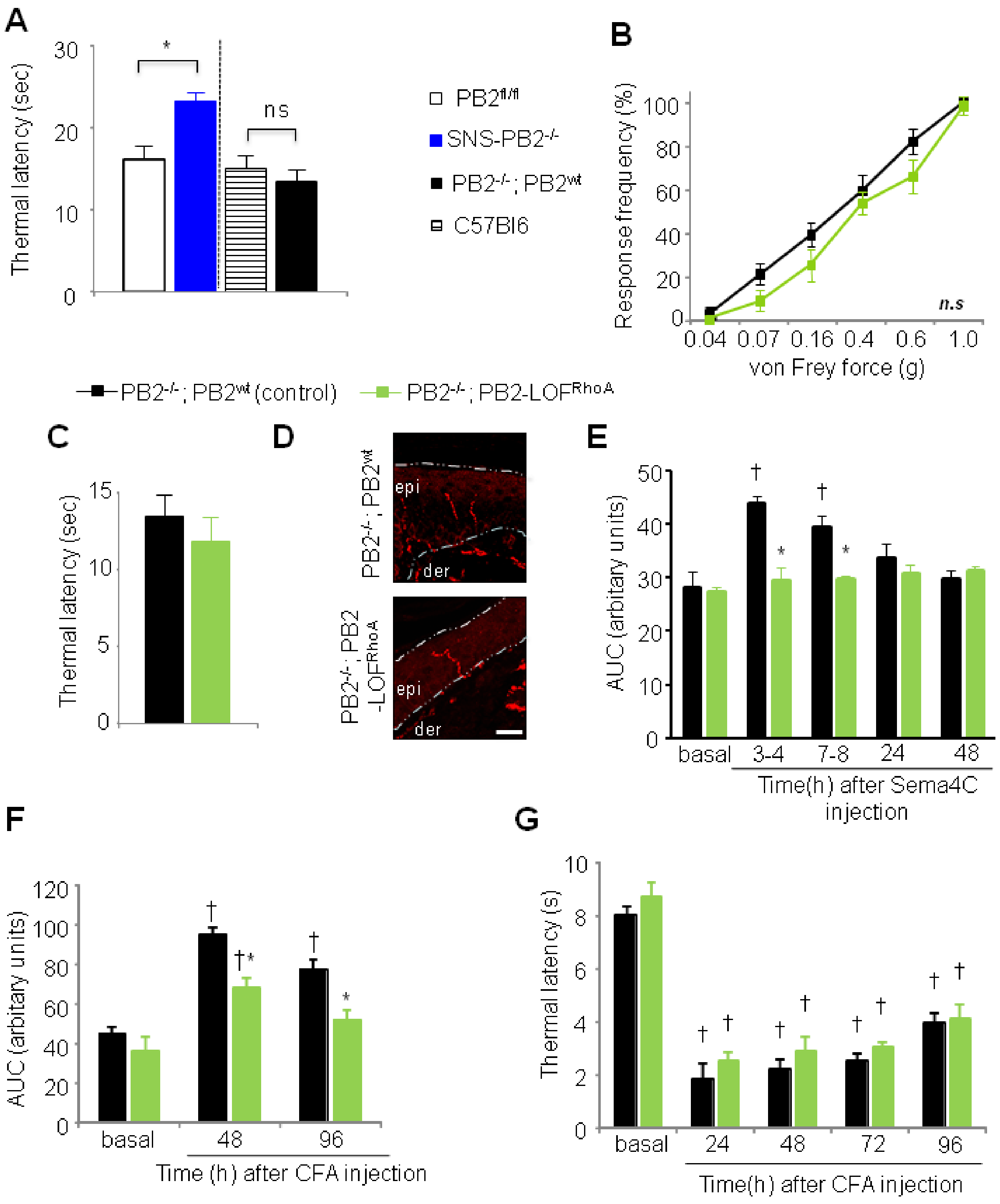

Supplementary Figure 7. Analysis of PB2$^{-/-}$; PB2-LOF^{RhoA} mice.

(A) Withdrawal latencies of PB2$^{fl/fl}$, SNS-PB2$^{-/-}$, PB2$^{-/-}$; PB2^{wt} and C57black6 mice measured with Hot Plate Test at 50°C. (B, C) Basal mechanical sensitivity (B) measured as response frequency to von Frey hair filaments and (C) withdrawal latency measured in Hot Plate Test at 50°C in PB2$^{-/-}$; PB2-LOF^{RhoA}, and in their corresponding PB2$^{-/-}$; PB2^{wt} controls (n = 6 mice/group) (D) Typical examples of the

density of peptidergic putative nociceptor endings in the hindpaw plantar skin in $PB2^{-/-}$; PB2-LOF^{RhoA}, and in their corresponding $PB2^{-/-}$; $PB2^{wt}$ controls. Scale bar represents 50 µm (n = 10-14 sections/group) (E) Analysis of Sema4C-induced mechanical hypersensitivity to plantar application of von Frey force in $PB2^{-/-}$; PB2-LOF^{RhoA} mice, and in their corresponding $PB2^{-/-}$; $PB2^{wt}$ controls. Integral responses to all graded mechanical von Frey stimuli (AUC) prior to and at 3-4, 7-8, 24 and 48 hours following 1ng Sema4C intraplantar injection are shown. (F) Analysis of inflammatory hypersensitivity, represented as integral responses to all graded mechanical von Frey stimuli (AUC) prior to and at 24, 48, 72 and 96 hours following hindpaw CFA injection in $PB2^{-/-}$; PB2-LOF^{RhoA} mice, and in their corresponding $PB2^{-/-}$; $PB2^{wt}$ controls. . (G) CFA induced thermal hypersitivity in $PB2^{-/-}$; PB2-LOF^{RhoA} mice and in their corresponding $PB2^{-/-}$; $PB2^{wt}$ controls. N = 5 mice/group for $PB2^{-/-}$;PB2-LOF^{RhoA} mice and n = 8 for control mice in all experiments E, F and G. ANOVA for random measures was performed in panels A and C; ANOVA for repeated measures was performed in panels B, E, F and G followed by Tukey´s test. Statistical significances ($P<0.05$) are indicated by † as compared to basal values within a group. Error bars, SEM.

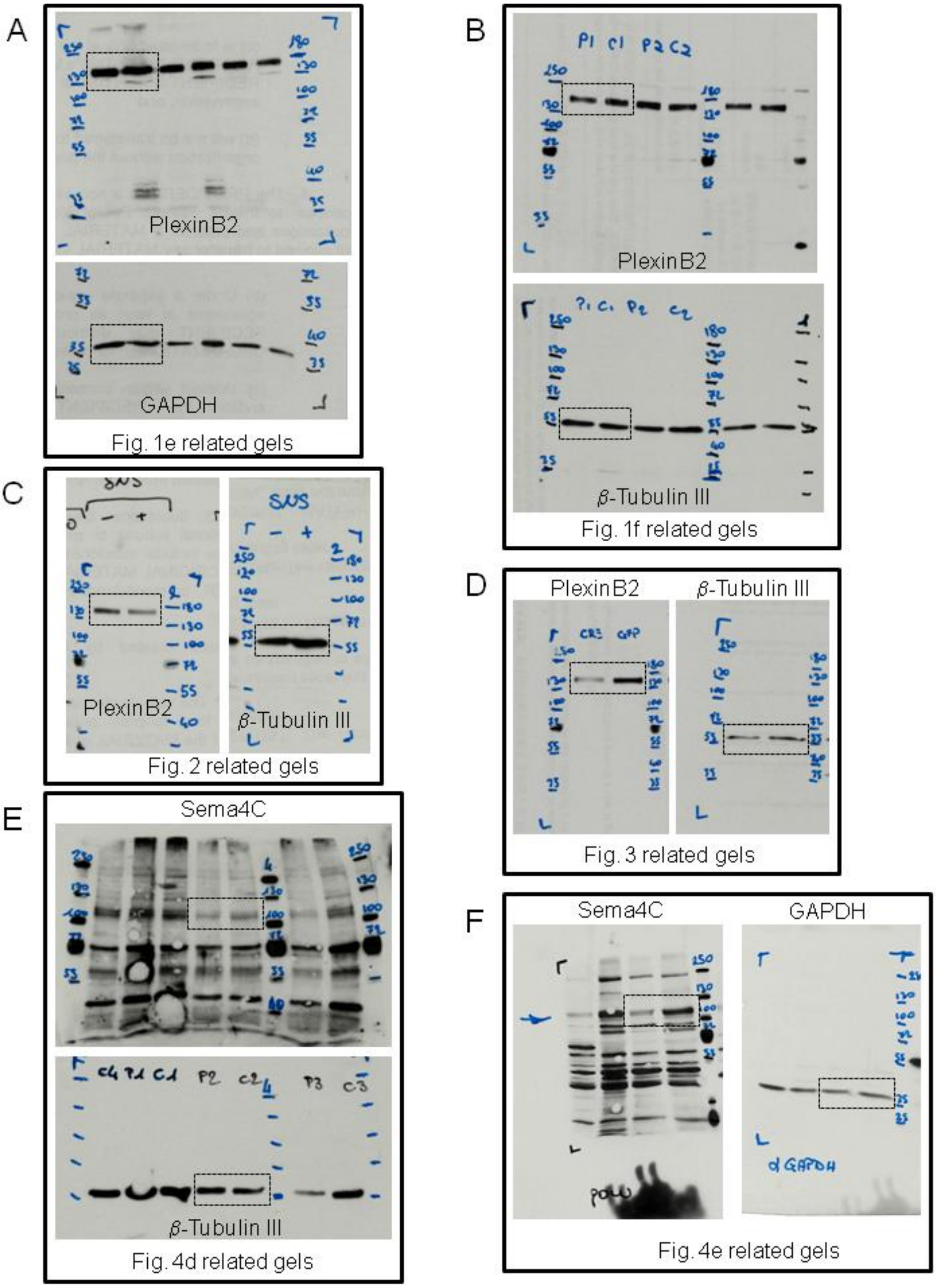
A
PlexinB2
GAPDH
Fig. 1e related gels
B
PlexinB2
β-Tubulin III
Fig. 1f related gels
C
PlexinB2
β-Tubulin III
Fig. 2 related gels
D
PlexinB2
β-Tubulin III
Fig. 3 related gels
E
Sema4C
β-Tubulin III
Fig. 4d related gels
F
Sema4C
GAPDH
Fig. 4e related gels

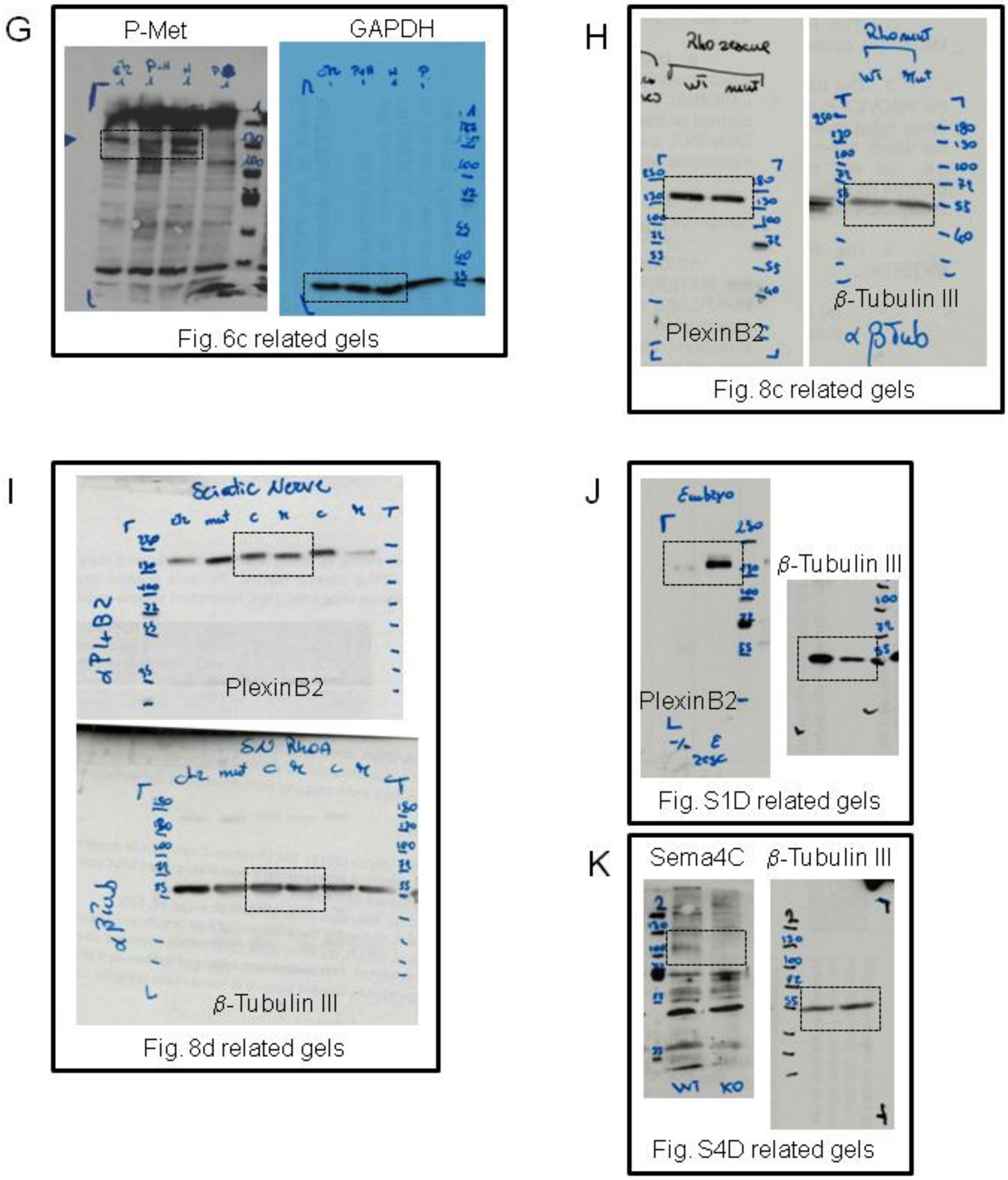

Supplementary Fig 8. Full size immunoblots.

Full-size immunoblots related to all western blot examples that are shown in all of the main and supplementary figures. Dashed boxes indicate regions shown in the corresponding figure.
